# Diverging trends in neurodevelopmental disorder diagnoses among children aged below 16 years in Kazakhstan (2014–2024): a national registry study

**DOI:** 10.3389/fpsyt.2026.1880419

**Published:** 2026-07-24

**Authors:** Amir Baikatov, Ayat Assemov, Dias Alimbetov, Almira Kustubayeva

**Affiliations:** 1 Brain Institute, Farabi Kazakh National University, Almaty, Kazakhstan; 2 Clinical Diagnostic Laboratory OLYMP, Astana, Kazakhstan; 3 Salidat Kairbekova National Scientific Center for Health Development of the Ministry of Health of the Republic of Kazakhstan, Astana, Kazakhstan; 4 Department of Biophysics, Biomedicine, and Neuroscience, Farabi Kazakh National University, Almaty, Kazakhstan

**Keywords:** ADHD, autism spectrum disorder, central Asia, epidemiology, healthcare registry data, intellectual disability, neurodevelopmental disorders

## Abstract

**Introduction:**

Neurodevelopmental disorders are increasingly straining health systems. However, how these disorders are distributed across populations, or whether their occurrence is increasing, is still not fully understood. This study examined registry-based prevalence, temporal trends, geographic variation, and associated systemic factors in a Central Asian setting.

**Methods:**

We analysed national electronic health-registry data from Kazakhstan for children aged ≤15 years from 2014 to 2024 using a repeated cross-sectional design. The register is a prevalent “on-the-register” stock registry, and the unit of analysis is the registry record per child-year; the pooled total of 214,904 records therefore represents child-year observations rather than unique children. Annual registry-based prevalence was calculated against official population denominators. Temporal trends were modelled with negative-binomial regression and summarized as annual percent change (APC) and segmented regression; regional differences and associated factors were examined with Bayesian hierarchical and mixed-effects models.

**Results:**

Among the 214,904 registered records, intellectual disability made up the largest share at 60.8%, followed by autism spectrum disorder at 20.9%, other developmental disorders at 17.1%, and attention-deficit/hyperactivity disorder at 1.1%. Registry-based ASD prevalence rose steeply from 16.8 to 190.5 per 100,000 (APC +27.0%/year), accelerating after the 2021 policy expansion. In contrast, other developmental disorders (−7.8%/year) and ADHD (−10.2%/year) declined, while intellectual-disability prevalence remained essentially stable (APC −0.7%/year, not statistically significant). The decline in the share of intellectual disability therefore reflects a compositional shift driven by the increase in ASD. Marked geographic differences were observed, with higher registry-based ASD prevalence in metropolitan areas and northern and eastern regions, together with strong differences related to the diagnosing specialist.

**Discussion:**

These patterns indicate that registered neurodevelopmental diagnoses in Kazakhstan are shaped not only by epidemiology but heavily by changing diagnostic practices, specialist availability, reimbursement policy, and the organization of services. Registry-based estimates should therefore be interpreted as measures of ascertainment rather than of true population prevalence. This study offers a national perspective on registry-based trends in developing regions and emphasizes the importance of systemic factors when planning services and estimating the true burden of these disorders.

## 1 Introduction

Neurodevelopmental disorders (NDDs) are a largely heterogeneous group of chronic neurological disorders that manifest early in life and alter the trajectory of typical nervous-system development, resulting in behavioral difficulties and reduced adaptability of the affected individual to societal and occupational contexts (1–3). In the fifth edition of the Diagnostic and Statistical Manual of Mental Disorders, the NDD classification includes intellectual disability (ID), autism spectrum disorder (ASD), and attention-deficit/hyperactivity disorder (ADHD), along with sparser or unspecified conditions. These disorders were also unified in the recently added “Mental, Behavioral and Neurodevelopmental disorders” chapter of the International Classification of Diseases, 11th revision. Despite an abundance of literature on specific NDDs, their prevalences vary drastically by region, time period, and data-collection methodology (4–10).

According to the GBD 2021 disease and injury burden analyses, approximately 235 million children under 15 years across 204 countries were affected with ASD, ADHD or ID (11), with estimated prevalences per 100,000 children of 857 for ASD, 1,662 for ADHD, and 1,626 for ID (12). Beyond limitations in awareness and service availability, the overlapping symptoms and high comorbidity rates inherent to NDDs complicate detection, diagnosis, care and treatment across the life course (10, 13–18).

Interpreting registry data from Kazakhstan requires understanding how these conditions are diagnosed and recorded. Throughout the study period, mental and behavioral disorders were diagnosed under the ICD-10 (ICD-11 has not yet been adopted in routine clinical practice). The diagnosis of ASD is the responsibility of child psychiatrists working in regional mental-health centers: a child with a suspected disorder is referred from primary care or from a Psychological–Medical–Pedagogical Consultation (PMPC), the psychiatrist establishes an ICD-10 diagnosis, and first-time diagnoses are entered into the state electronic health information system and the statistical register within a few working days. ADHD, by contrast, is frequently first encountered by pediatricians or family physicians and is not subject to the same mandatory psychiatric-registration pathway. These features directly affect detection, case registration and the apparent dynamics of prevalence.

Research on NDDs in Central Asia remains sparse, but a national evidence base is emerging for Kazakhstan. A regional and national report described a steep rise in registered childhood and atypical autism between 2016 and 2022 with little change in broader NDD categories (19), and a separate national study using the same electronic health system reported rising ASD incidence (12.8 to 26.6 per 100,000) and period prevalence in children over 2014–2021 (20). Population surveys indicate limited public awareness of ASD (21), while studies of clinicians document knowledge gaps and a shortage of trained specialists (22, 23). Caregivers report difficulties accessing health care and inclusive education, and elevated stress, anxiety and depression associated with stigma and misinformation (24–26). Economic and policy evaluations have begun to quantify the labor-market and productivity burden of ASD on families and the state (27). Against this background, in 2021 childhood autism, atypical autism and Asperger syndrome were recognized for disability and subsidy purposes, a change temporally aligned with accelerated ASD registration in children (19).

Analysis of the registry profile of children in Kazakhstan thus provides a valuable perspective on diagnostic trends in mental conditions emerging from a rigid post-Soviet context (21). This study aimed to describe trends in registered NDD cases among children in Kazakhstan from 2014 to 2024, focusing on their demographic composition and diagnostic distribution across the country’s regions, for medical practitioners, researchers and policymakers.

## 2 Materials and methods

### 2.1 Setting

This registry-based study was conducted in the Republic of Kazakhstan using routinely collected health-care data from the national health information infrastructure under the Ministry of Health. The study is reported in accordance with the RECORD extension to the STROBE statement for studies using routinely collected health data; a completed RECORD checklist is provided as **Supplementary Material**.

### 2.2 Data sources

#### 2.2.1 National health registry extraction (numerators)

The National Scientific Center for Healthcare Development named after Salidat Kairbekova provided an anonymized extract of individual-level register records. The extract included the year of registry entry, the primary ICD-10 diagnosis code, age at assessment, diagnosis date, sex, region of residence, urban/rural residence, citizenship, ethnicity, a child placement/educational indicator, and the specialty of the diagnosing clinician.

To support transparent appraisal of the data (RECORD), we summarize how records enter the register. A diagnosis is established within the state service network described above and recorded in the national electronic health information system; reporting is mandatory for state healthcare organizations nationwide. Diagnoses that confer disability status or state subsidies are issued or confirmed within the state system, so they are captured by the register; purely private assessments that are not entered into the state system, and educational evaluations that do not result in a clinical ICD-10 entry, are not captured. The register is maintained as a prevalent (“on-the-register”) system: each annual extract lists the children under active observation for a registered NDD in that calendar year, so a child who remains registered contributes one record in each year of registration. The anonymized extract does not contain a personal identifier valid across yearly files; we verified that the per-file record identifier is reused across years and is associated with inconsistent birth dates and sex, so records cannot be de-duplicated or linked at the individual level across years.

#### 2.2.2 Official population statistics (denominators)

Publicly available data from the Bureau of National Statistics of the Agency for Strategic Planning and Reforms of the Republic of Kazakhstan (28) was accessed for annual population denominators as regional population counts for children aged 0–15 years (≤15 years) for the corresponding calendar years. Year-end counts were used based on availability.

### 2.3 Study design

A repeated cross-sectional study of annual registry information was conducted to characterize national time trends and regional variation in registered NDDs among children. Analyses were conducted at the level of anonymized registry records within each calendar year, and registry extracts were arranged by year. The unit of analysis is therefore the registry record per child-year, not the unique child. Annual administrative records represent registry-based (point) prevalence rather than cumulative incidence. Because individual children cannot be linked across years (§2.2.1), the pooled count of records is a count of child-year observations: the 214,904 records analyzed correspond to 68,384 distinct registry identifiers (≈3.1 records each), and should not be interpreted as 214,904 unique individuals. Increasing annual counts therefore reflect changes in the registered (ascertained) burden rather than a direct measure of newly affected children.

### 2.4 Study population

We studied children aged ≤15 years (i.e., under 16 at assessment). This age band was chosen because the focus is childhood-onset NDDs in the pediatric population; the official QazStat denominators are published for the 0–15 child age band, enabling internally consistent population-based rates; the band aligns with GBD pediatric NDD reporting used for international comparison; and it corresponds to the pediatric service network that feeds the register. Records linked to official denominators with a valid primary diagnostic code within the case definition and a non-missing age supporting the inclusion requirement were considered eligible. Records without a verified diagnosis date were excluded from analytic models that required diagnosis-year information.

### 2.5 Case definition and classification

NDDs were defined using primary ICD-10 diagnostic codes and grouped into four prespecified categories: (I) ASD (F84.*); (II) ADHD (F90.*); (III) ID (F70–F79); and (IV) other developmental disorders. The complete set of ICD-10 codes observed in each group, with counts and within-group percentages, is given in **Supplementary Table 2**. In brief, “other developmental disorders” comprises specific developmental disorders of speech/language (F80.0–F80.9), scholastic skills (F81.0–F81.9) and motor function (F82), mixed specific developmental disorders (F83), other and unspecified disorders of psychological development (F88, F89), and tic disorders (F95.0–F95.9) — 23 distinct codes, dominated by F83 (47.5%) and F80.0 (30.5%). For transparency, the ASD group as coded includes small numbers of Rett syndrome (F84.2, n=100) and other childhood disintegrative disorder (F84.3, n=48); a sensitivity analysis excluding these codes did not alter any conclusion.

### 2.6 Measures

Sociodemographic characteristics included sex, region of residence, urban versus rural residence, age at registry contact/assessment and, where available, age at diagnosis. Ethnicity and citizenship were summarized as reported, with nonsensical or absent entries coded as “unknown/not reported.” Diagnosing clinicians were categorized into interpretable provider groups (psychiatrist, neurologist, pediatrician, general practitioner/family physician, psychologist, mid-level practitioner, other/unknown), and child placement/educational status into child-relevant categories (home care, preschool, school-age student, out-of-home care, other).

#### 2.6.1 Outcomes

Registry-based prevalence was computed as the number of registry records per calendar year divided by the corresponding official child (≤15 y) population, expressed per 100,000, overall and by diagnostic category and region. As a record-level proxy for first-recorded diagnoses, we additionally counted records whose recorded diagnosis year equaled the registry year, expressed per 100,000 using the same denominators. Because individual children cannot be linked across years, this measure is defined strictly at the record level and is interpreted as a registry-based approximation of newly recorded diagnoses, not as person-level population incidence.

### 2.7 Regional harmonization and administrative changes

A single standardized approach harmonized region names across government demographic data and the register. Numerator–denominator merges did not include region-year combinations that did not exist historically owing to administrative reforms (notably the 2018 and 2022 reorganizations).

### 2.8 Statistical analysis

Descriptive summaries of the distribution of sex, age, region, citizenship, ethnicity, clinician specialty and child placement/educational group were generated by calendar year and diagnostic category. Annual registry-based prevalence was computed by diagnostic category at national and regional levels.

National temporal trends. Time trends in national annual case counts were modelled with negative-binomial (NB2) regression using calendar year (centered at 2014) as the predictor and the log of the official child population as an offset, yielding incidence-rate ratios (IRR) per one-year increase with 95% confidence intervals. NB was preferred over Poisson because the counts were overdispersed: the Poisson Pearson dispersion statistic was 13.7 (ASD), 18.1 (ID) and 21.5 (other developmental), and 1.6 for ADHD; a likelihood-ratio comparison favored NB for the overdispersed series. To quantify trends explicitly, we computed the annual percent change (APC) from a log-linear model of registry-based prevalence and fitted a segmented (joinpoint-style) regression with breakpoints fixed at the 2019 PMPC restructuring and the 2021 ASD policy expansion, reporting period-specific APCs.

Composition models. To investigate whether between-region variation in diagnostic make-up was associated with region-year characteristics, Bayesian hierarchical binomial-logit models with region-level random intercepts were fitted to region-year-aggregated data. Proportion-based predictors were scaled per 10 percentage points and centered; odds ratios with 95% credible intervals (CrI) are reported. Models were fitted in PyMC by Hamiltonian Monte Carlo (NUTS) with weakly-informative priors (Intercept ~ Normal(0, 1); coefficients ~ Normal(0, 0.30); region SD ~ HalfNormal(0.40); region effects ~ Normal(0, σ_region)), using 2 chains of 1,500 warm-up plus 1,500 posterior draws each (3,000 posterior draws), target acceptance 0.97 and a fixed seed; region-year cells with fewer than 10 records were excluded. Convergence was assessed by R-hat (≤1.01 for reported parameters) and bulk/tail effective sample sizes, and model adequacy by posterior predictive checks; R-hat and ESS are reported alongside each estimate in the **Supplementary Material**.

Individual-level ASD model. To examine record-level correlates of an ASD classification, a mixed-effects logistic model was fitted with the outcome “record coded ASD vs other NDD,” fixed effects for registry year, age at diagnosis, sex, child placement/educational status and urban/rural residence, and random intercepts for region and diagnosing-specialist group. For tractability, models used balanced case–control sampling (all index-diagnosis records as cases; non-index records sampled at a fixed ratio — ASD and other developmental 1:5, ADHD 1:10, ID 1:2 with cases capped at 50,000; for ASD the available controls were fewer than the 1:5 target, so essentially all controls were used). No observation weighting was applied. As a sensitivity analysis we repeated the sampling 50 times with independent seeds and compared the estimates with a full-data fit; results were stable (**Supplementary Table 3**). Estimated odds ratios describe within-registry relative odds, not population-prevalence odds, and their narrow intervals reflect the large within-registry sample size.

Regional co-trending. Spearman rank correlations between region-specific annual counts of ASD subdiagnoses (F84.x) and ungrouped developmental diagnoses (F80–F89; F95) were calculated and summarized across regions using median ρ and median p-values. These correlations are ecological and are interpreted as hypothesis-generating, not as evidence of individual diagnostic transitions.

### 2.9 Handling of missing values

Geographic and age-related variables showed near-complete coverage. Ethnicity was missing or unclear in 37.0% of records and citizenship was absent in 12.0%; 98.7% of entries did not record comorbidities, which were therefore not analyzed. Provider specialty was largely complete (1.1% unknown). Regression models used complete-case analysis for included variables while retaining “unknown/not reported” categories for descriptive reporting; diagnosis-date analyses were limited to records with valid dates within the study period.

### 2.10 Software and reproducibility

All data processing, analyses and figure generation were performed in Python with standard scientific libraries for statistical modeling, Bayesian inference and geospatial analysis. Processed datasets and analysis scripts sufficient to reproduce the findings, including the APC, segmented-regression, overdispersion and sensitivity analyses, are available in the project repository.

## 3 Results

### 3.1 Sample characteristics and diagnostic grouping

The analytic dataset comprised 214,904 registry records (child-years) for children aged ≤15 years over 2014–2024, corresponding to 68,384 distinct registry identifiers; most records (73.6%) carried a diagnosis date predating the registry year, consistent with a prevalent-stock register. Across the four diagnostic groups (**Table 1**; **Supplementary Table 1**), grouped counts were primarily driven by a small number of high-frequency primary diagnoses. In the ASD group, childhood autism (F84.0; n=27,053) and atypical autism (F84.1; n=15,918) together accounted for 42,971 of 45,010 (95.5%) records; F84.0 was most often diagnosed by a psychiatrist (56.7%) and most frequent in Astana City (29.1%), while F84.1 was most often diagnosed by a psychiatrist (43.5%) and most frequent in Almaty City (24.6%). In the ADHD group, predominantly inattentive (F90.0; n=1,208) and predominantly hyperactive/impulsive (F90.1; n=1,175) diagnoses comprised 2,383/2,413 (98.8%) records, with family physician the most frequent diagnosing specialist. In the other-developmental group, mixed specific developmental disorders (F83; n=17,441) and phonological disorder (F80.0; n=11,206) together comprised 28,647/36,732 (78.0%).

**Table 1:** Characteristics of children aged ≤15 years with registered neurodevelopmental disorders in Kazakhstan (aggregated diagnostic groups).

Characteristic	Attention-deficit/hyperactivity disorder (ADHD)	Autism spectrum disorder (ASD)	Intellectual disability (ID)	Other developmental disorders
N (children)	2,413	45,010	130,749	36,732
% of total sample	1.12	20.94	60.84	17.09
Age at diagnosis, years [median (IQR)]	7.2 (5.7–8.8)	5.8 (4.6–7.5)	7.5 (5.6–10.0)	6.6 (4.7–8.3)
Age at assessment, years [median (IQR)]	10.0 (8.0–12.0)	8.0 (6.0–10.0)	11.0 (9.0–13.0)	9.0 (7.0–12.0)
Year of diagnosis [median (IQR)]	2016 (2013–2019)	2020 (2018–2022)	2017 (2013–2020)	2016 (2013–2019)
Male, %	81.7	78.2	61.3	67.1
Kazakhstan citizenship, %	80.1	87.4	88.8	86.6
Urban residence, %	62	45	47.3	66.6
Most frequent primary dx (share, %)	ADHD, predominantly inattentive (F90.0) (50.1%)	Childhood autism (F84.0) (60.1%)	Mild intellectual disability, without behavioral impairment (F70.0) (22.0%)	Mixed specific developmental disorders (F83) (47.5%)
Most frequent specialist (share, %)	Family physician (38.1%)	Psychiatrist (51.6%)	Psychiatrist (40.4%)	Family physician (29.7%)
Most frequent region (share, %)	Turkistan (32.5%)	Astana City (21.4%)	Almaty City (9.1%)	Turkistan (30.6%)
Most frequent child placement (share, %)	Out-of-home care (60.8%)	Home care (40.7%)	Out-of-home care (41.7%)	Out-of-home care (49.0%)
Most frequent ethnicity (share, %)	Kazakh (40.7%)	Kazakh (72.8%)	Kazakh (70.6%)	Kazakh (55.9%)

Summary of registry records and composition for ADHD, ASD, intellectual disability, and other developmental disorders, including age at diagnosis and assessment [median (IQR)], year of diagnosis [median (IQR)], sex distribution, citizenship, urban residence, and the most frequent primary ICD-10 diagnosis, diagnosing specialist, region, child placement/educational status, and ethnicity (with category share, %).

### 3.2 National temporal trends in registry-based prevalence and first-time diagnoses

**Figure 1:**
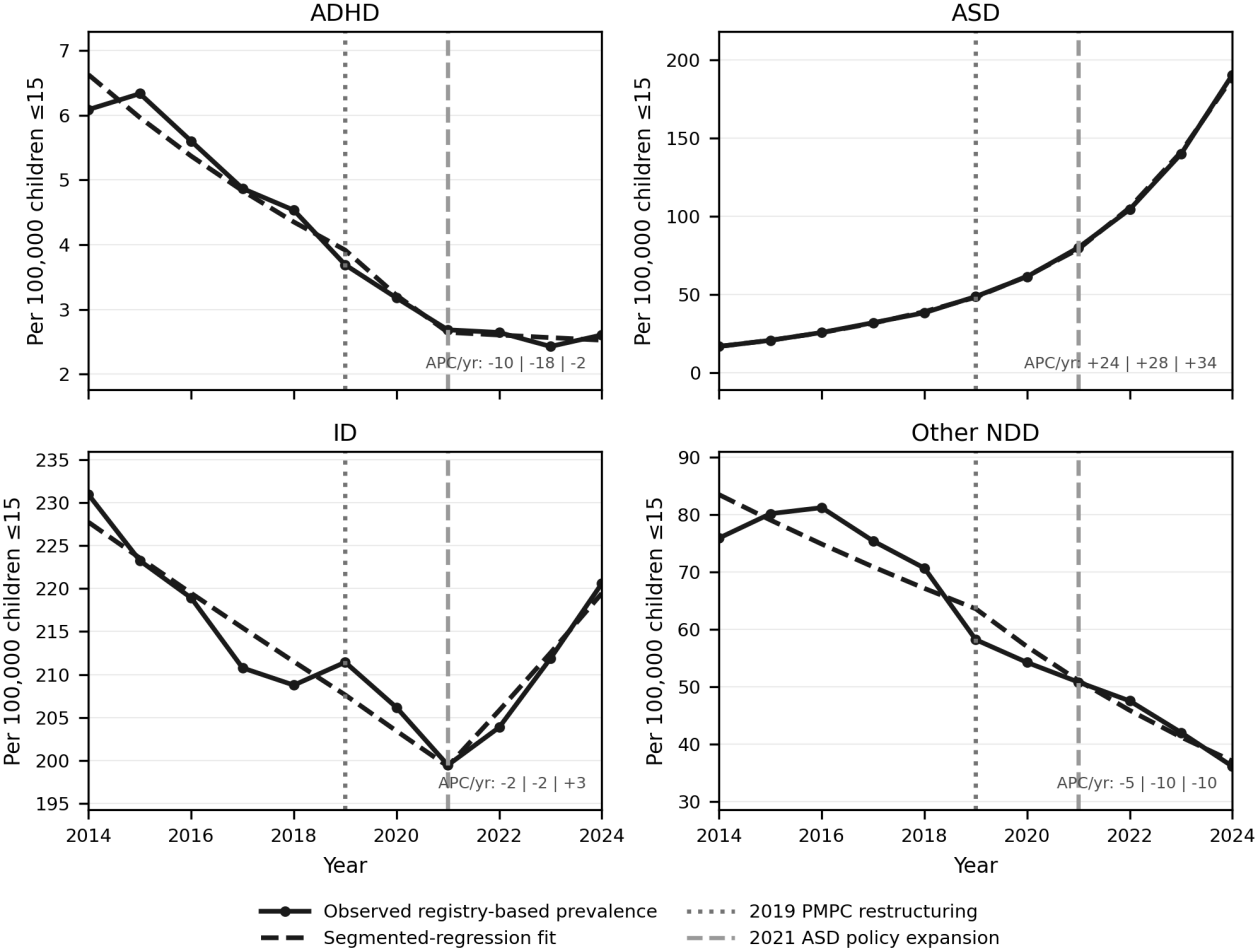
National trends in registry-based prevalence and first-time diagnoses of neurodevelopmental disorders among children aged ≤15 years, Kazakhstan, 2014–2024. Four panels show annual registered prevalence (solid line) per 100,000 children aged ≤15 years and segmented-regression slopes (dashed line), for ADHD **(A)**, ASD **(B)**, intellectual disability **(C)**, and other developmental disorders **(D)**. Rates were calculated using national population denominators for ages ≤15 years. Vertical reference lines indicate the 2019 restructuring of Psychological–Medical–Pedagogical Consultations (PMPCs) and the 2021 ASD-related policy expansion. Annual percent change is reported for each panel.

National time-series analyses showed marked divergence across diagnostic groups (**Figure 1**). Registry-based ASD prevalence increased steeply and persistently, from 16.8 to 190.5 per 100,000 (APC + 27.0%/yr; 95% CI 25.4 to 28.7; NB IRR 1.27 per year, 1.26–1.29). Other developmental disorders declined (75.9 to 36.1 per 100,000; APC −7.8%/yr; IRR 0.92, 0.91–0.93) and ADHD declined from an already low level (6.1 to 2.6 per 100,000; APC −10.2%/yr; IRR 0.90, 0.88–0.91). Registry-based ID prevalence was essentially stable (230.9 to 220.6 per 100,000; APC −0.7%/yr, 95% CI −1.5 to 0.1, not significant; IRR 0.99, 0.99–1.00); the fall in the share of ID within the register therefore reflects a compositional shift produced by the ASD increase rather than a decline in ID prevalence. Segmented regression indicated that the ASD increase accelerated across periods (+23.5%/yr in 2014–2019, +28.0%/yr in 2019–2021, +33.7%/yr in 2021–2024), consistent with a policy-linked rise after 2021. Patterns based on the record-level new-diagnosis proxy broadly mirrored these findings. A transient spike coinciding with the 2019 PMPC restructuring was more apparent for ADHD and other developmental disorders than for ASD, which continued to rise after 2021.

### 3.3 Geographic heterogeneity and regional clustering

**Figure 2:**
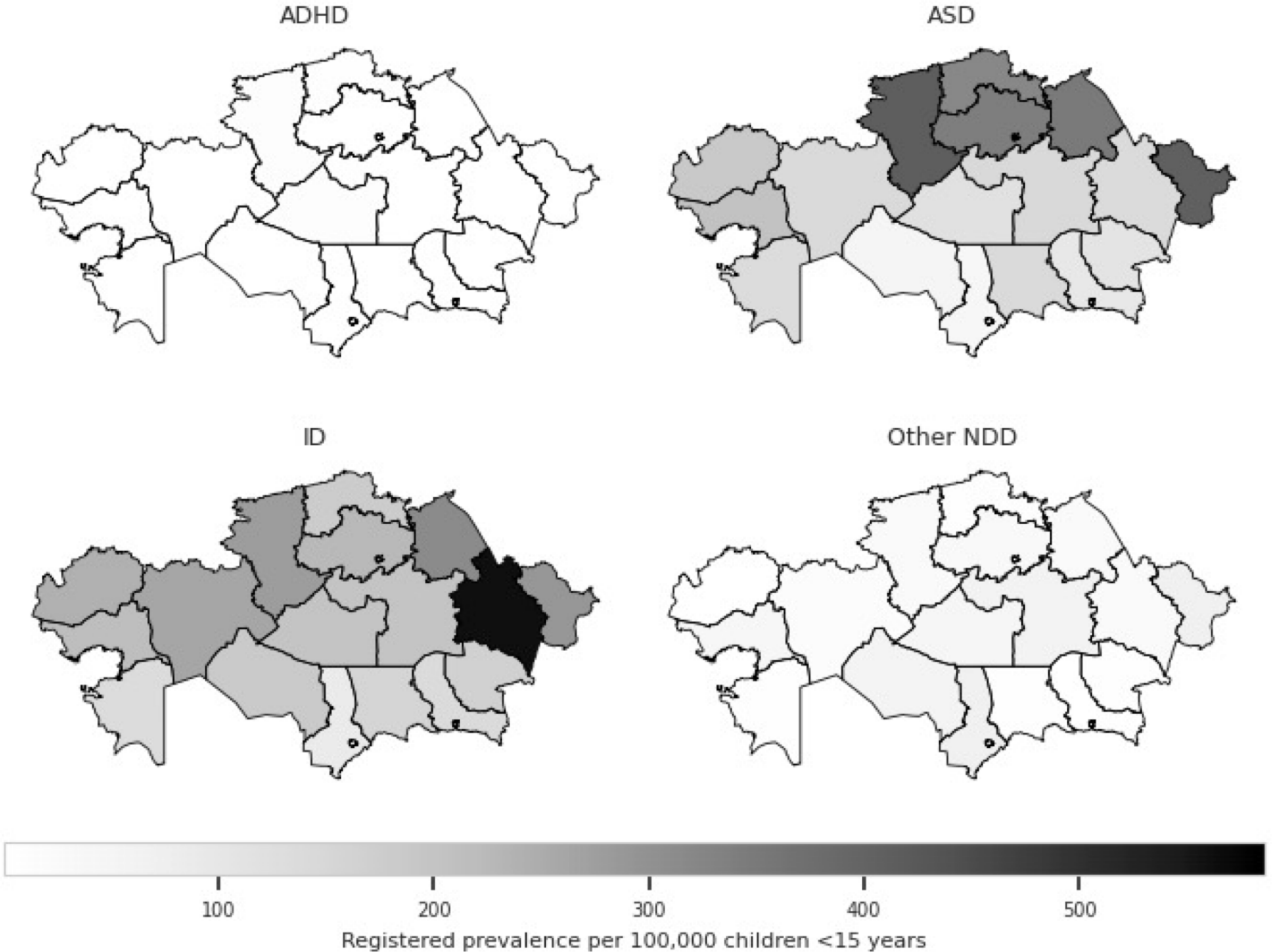
Regional variation in registered prevalence of neurodevelopmental disorders among children aged ≤15 years, Kazakhstan, 2024. Choropleth maps display registered prevalence per 100,000 children aged ≤15 years by region for ADHD **(A)**, ASD **(B)**, intellectual disability **(C)**, and other developmental disorders **(D)** in 2024. Shading indicates higher registry-based prevalence.

By the latest available data (2024), registry-based ASD prevalence was highest in major metropolitan and northern regions, most prominently Astana City, whereas ID prevalence remained high across multiple regions with pronounced peaks; ADHD prevalence was uniformly low (**Figure 2**). Region-by-year heatmaps showed that national trends were not spatially uniform (**Supplementary Figure 1**). Because the register captures ascertainment rather than occurrence, these metropolitan peaks most plausibly reflect the concentration of child psychiatrists, PMPCs and specialized diagnostic centers, shorter referral paths and greater awareness in Astana and Almaty, rather than true regional differences in the occurrence of NDDs (see §4.1).

### 3.4 Determinants of registry-based prevalence in composition models

**Figure 3:**
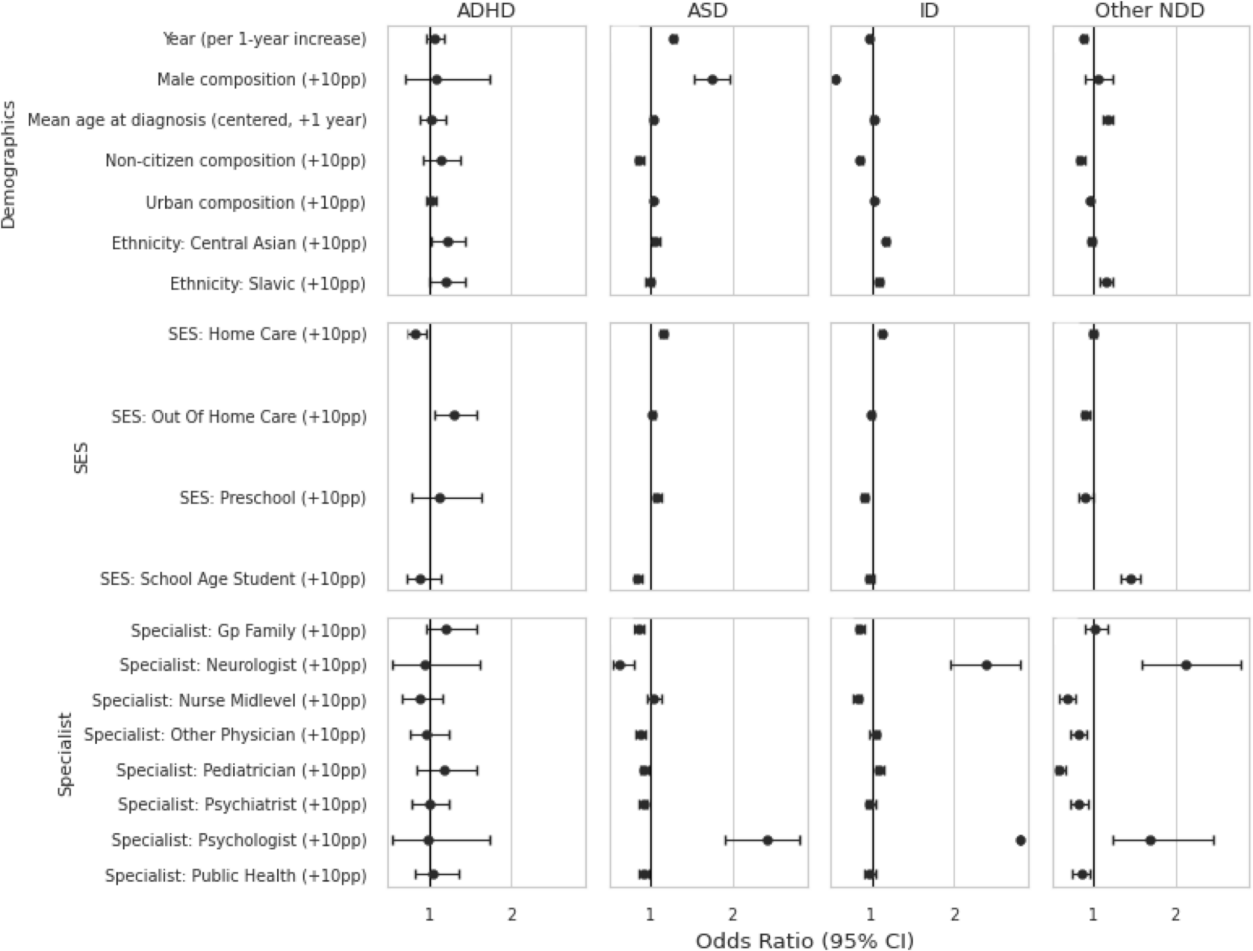
Factors associated with registry-based prevalence of neurodevelopmental disorders: multivariable model estimates across diagnostic groups. Forest plot summarising adjusted associations between registry year, demographic composition, child placement/educational composition, and diagnosing specialist composition with disorder-specific registry-based prevalence for ADHD **(A)**, ASD **(B)**, intellectual disability **(C)**, and other developmental disorders **(D)**. Points indicate effect estimates (odds ratios) and horizontal bars represent 95% credible/confidence intervals; the vertical reference line marks the null value (1.0).

In region-/stratum-level composition models (**Supplementary Table 2b**; **Figure 3**), registry year showed a strong positive association for ASD (OR 1.28 per year; 95% CrI 1.25–1.31) but negative associations for ID (OR 0.97; 0.95–0.99) and other developmental disorders (OR 0.89; 0.87–0.92); the ADHD year effect was smaller (OR 1.07; 0.96–1.19). Higher male composition was associated with ASD (OR 1.74 per +10 pp; 1.52–1.97) and inversely with ID (OR 0.52; 0.47–0.58). Higher neurologist composition was associated with higher ID (OR 2.38; 1.95–2.81) and other developmental (OR 2.12; 1.59–2.79) but lower ASD (OR 0.62; 0.48–0.80); higher psychologist composition was positively associated with all groups, particularly ID (OR 5.37; 4.46–6.41). These specialist–diagnosis associations should be read as reflections of referral pathways and the organization of services — which children are seen by, and which providers are authorized to assign, each diagnosis — rather than as causal effects of specialist practice.

### 3.5 Individual-level multilevel model for ASD diagnosis within the registry

**Figure 4:**
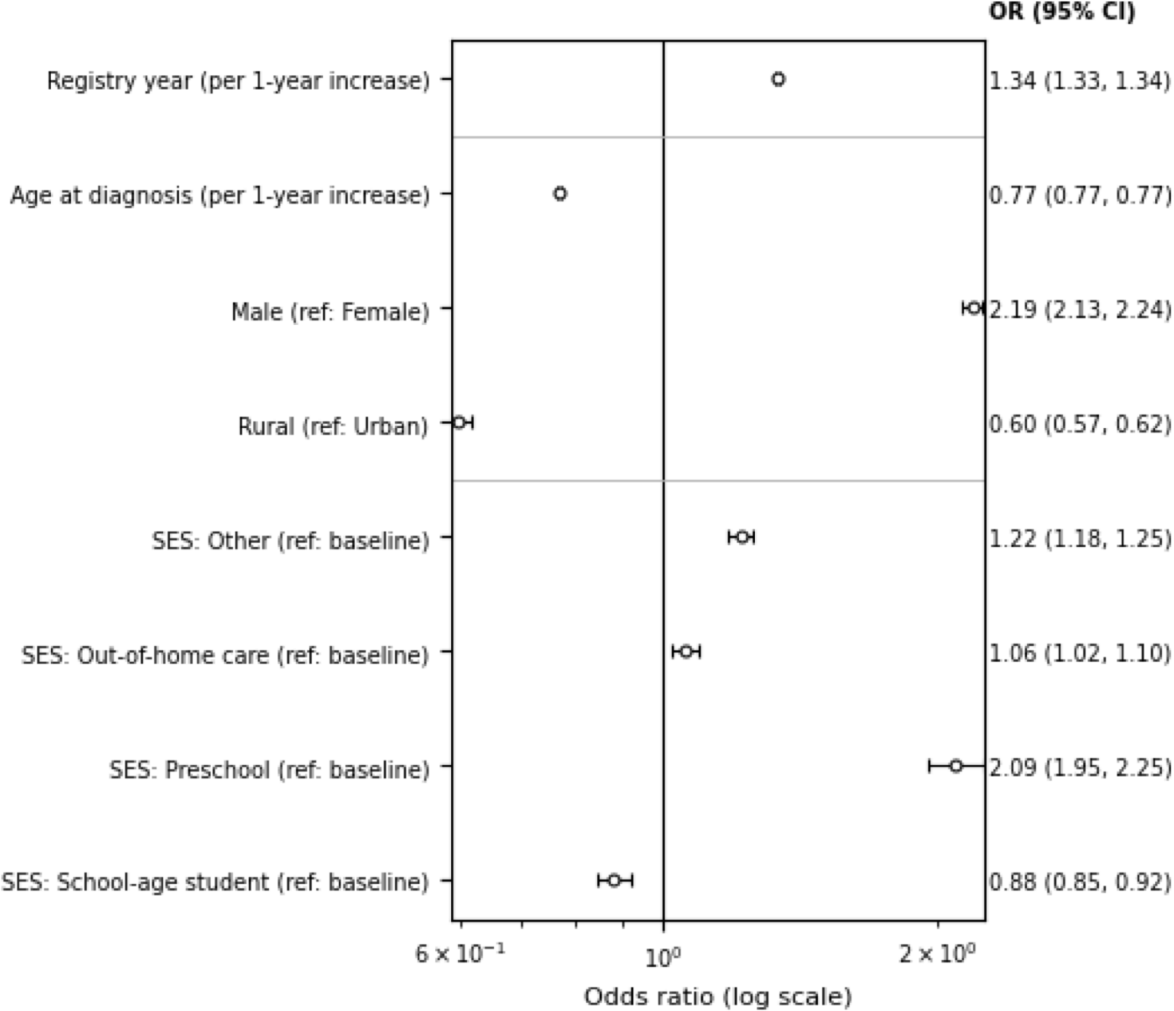
Autism spectrum disorder: adjusted associations from the individual-level multivariable model (mixed-effects logistic model). Forest plot of adjusted odds ratios (log scale) from an ASD mixed-effects logistic model. Predictors include registry year (per 1-year increase), age at diagnosis (per 1-year increase), sex (male vs female), residence (rural vs urban), and child placement/educational status categories (relative to the baseline category). Points indicate posterior means and horizontal bars indicate 95% credible intervals.

In the individual-level mixed-effects logistic model for ASD (**Supplementary Table 2c**; **Figure 4**), the odds that a record was classified as ASD (vs other NDD) increased with later registry year (OR 1.34 per year; 95% CrI 1.33–1.34) and were higher in males (OR 2.19; 2.13–2.24). Older age at diagnosis was associated with lower odds (OR 0.77 per year; 0.77–0.77) and rural residence with lower odds than urban (OR 0.60; 0.57–0.62); preschool status showed the largest positive association (OR 2.09; 1.95–2.25). These estimates describe within-registry relative odds rather than population-level prevalence odds, and their narrow intervals reflect the large within-registry sample size; convergence was satisfactory (R-hat ≤ 1.01). A subsampling sensitivity analysis (50 resamples vs full-data fit) confirmed that the estimates were not materially affected by case–control subsampling (**Supplementary Table 3**).

### 3.6 Regional co-trending of ASD subdiagnoses and other developmental diagnoses

The strongest inverse regional co-trending patterns appeared between ASD subdiagnoses and certain developmental disorders (**Supplementary Table 4**): specific reading disorder showed inverse correlations with childhood autism (median ρ = −0.719, p = 0.034) and atypical autism (ρ = −0.724, p = 0.021), each based on nine regional time series. These are ecological associations across regions and years; because individual children cannot be linked over time, they are compatible with — but do not demonstrate — diagnostic redistribution, and estimates based on few contributing regions should be interpreted cautiously.

## 4 Discussion

This national registry analysis shows a divergence in recorded diagnostic trajectories for pediatric NDDs in Kazakhstan: ASD registrations increased rapidly over 2014–2024, while other developmental disorders and ADHD declined and ID remained common. Three findings anchor interpretation: (i) the ASD increase is large and model-robust (**Figures 1**, **3**, **4**; APC + 27%/yr); (ii) it is geographically clustered rather than uniform (**Figure 2**); and (iii) recorded diagnostic composition is strongly associated with who assigns diagnoses (**Figure 3**) — consistent with a health system that is still consolidating diagnostic pathways and norms for NDDs.

### 4.1 Interpreting the ASD rise: ascertainment and possible redistribution

The increase in children with an ASD diagnosis is most consistent with improved recognition, diagnosis and service availability, together with greater awareness and declining stigma (19). Several features of the data support an ascertainment interpretation. The increase was concentrated in metropolitan and northern regions and among urban residents; higher registry-based ASD prevalence in Astana and Almaty is most plausibly explained by the concentration of child psychiatrists, PMPCs and diagnostic centers and by easier access and awareness, rather than by true regional differences in occurrence — a pattern repeatedly reported internationally, where diagnostic availability drives ascertainment (12). The falling age at diagnosis and the larger share of preschool-age children are also compatible with improving diagnostic efficiency. The negative ecological correlations between ASD subtypes and certain developmental codes, together with the stable ID prevalence but falling ID share, are compatible with diagnostic redistribution; however, because individual children cannot be linked across years, we cannot observe diagnostic transitions and do not claim case-for-case substitution. The 2021 recognition of childhood and atypical autism for disability/subsidy purposes is temporally aligned with the post-2021 acceleration in ASD registration, particularly in the capital. Overall, these findings are better viewed as improved visibility of ASD than as a true epidemic, although diagnostic gains must be matched by support capacity to avoid downstream harms (1, 24, 25).

### 4.2 Intellectual disability: stable prevalence, declining share

Our analyses clarify that registry-based ID prevalence per 100,000 was essentially stable over the period (APC −0.7%/yr, ns), even as the share of ID within the register fell. The apparent “decline” of ID is therefore largely a compositional consequence of the ASD surge rather than a fall in ID occurrence or registration. Consistent with this, regional heterogeneity and the limited availability of diagnosing specialists appear to constrain registered prevalence, and the tendency of practitioners to omit comorbidities in routine entries may further blur the ID–ASD boundary (16). These factors may also explain why the expected association of higher ID with lower socioeconomic metrics was not confirmed in our data (12).

### 4.3 The near-absence of ADHD: under-recognition and pathway mismatch

Registry-based ADHD prevalence was very low and declined (6.1 to 2.6 per 100,000; APC −10.2%/yr) — far below international estimates on the order of 1–5% — which we interpret as substantial under-recognition and under-registration rather than a genuinely low occurrence. Several Kazakhstan-specific factors are relevant. Unlike ASD, ADHD is not subject to a mandatory psychiatric-registration pathway; first-line identification is often by family physicians (the most frequent diagnosing specialist for ADHD in our data) rather than psychiatrists; standardized screening instruments are seldom used; stigma and limited awareness persist; and milder, higher-functioning presentations are the most easily missed in routine services. Differentiating clinically significant inattentive or hyperactive symptoms from normative behavioral variability may also be more difficult in a high-stimulation contemporary environment (29). The comparatively larger registration around the 2019 mandatory pre-school assessment, when the PMPC structure shifted from purely diagnostic to mandatory allocative assessment (30), is consistent with ascertainment-driven counts. Emerging awareness initiatives may increase recognition in future years. These observations are consistent with the established underestimation of ADHD in registry studies relative to survey and clinical designs (9).

### 4.4 Strengths and limitations

Strengths include large national coverage across a long time window, a consistent diagnostic grouping framework, and the combination of national trend models with regional and individual-level multilevel analyses.

Limitations are inherent to a prevalent administrative register. First, the register captures only children who reach state diagnostic services, introducing selection/ascertainment bias, so registry-based prevalence is a lower bound on true prevalence. Second, the anonymized extract cannot be linked at the individual level, precluding estimation of incidence and of diagnostic transitions; most annual records are carried-over prevalent cases. Third, substantial geographic variation in the availability of specialists, PMPCs and diagnostic centers and in referral pathways shapes the observed regional patterns. Fourth, routine diagnostic coding without standardized validation may vary across regions and clinicians, introducing information bias, including non-uniform use of ASD subtypes and developmental codes. Fifth, policy and reimbursement changes — the 2019 PMPC restructuring and the 2021 recognition of ASD for disability/subsidy purposes — may change registration independently of disease occurrence. Finally, private assessments not entered into the state system, and purely educational evaluations, are not captured. These factors are central to interpreting the findings, which describe registered diagnoses rather than the true occurrence of NDDs.

## 5 Conclusion

This study provides an overview of trends and the composition of registered NDDs among children aged ≤15 years in Kazakhstan. The national register shows a steep, geographically clustered rise in ASD registration, a compositional decline in the share of intellectual disability against an essentially stable ID prevalence, and persistent under-registration of ADHD. These changes are most consistent with evolving diagnostic practice, specialist availability and policy rather than with abrupt changes in the underlying occurrence of disorders, and registry-based estimates should be interpreted accordingly. Further development should include decentralization of specialists, updating of educational pathways, and destigmatization of NDDs to improve diagnosis and support.

## Data Availability

The datasets presented in this study can be found in online repositories. The names of the repository/repositories and accession number(s) can be found in the article/**Supplementary Material**.
