# Supplementary material for: Diverging trends in neurodevelopmental disorder diagnoses among children aged below 16 years in Kazakhstan (2014–2024): a national registry study
Source: Front Psychiatry. 2026 Jul 24;17:1880419. doi: 10.3389/fpsyt.2026.1880419 (PMC13448550; doi:10.3389/fpsyt.2026.1880419)
Supplement: Supplementary file 1 [file SupplementaryFile1.docx]

***Supplementary Material***

# 1 Supplementary Figures and Tables

## 1.1 Supplementary Figures

**Supplementary Figure S1. Regional time series of registered prevalence of neurodevelopmental disorders among children aged ≤15 years, Kazakhstan, 2014–2024. Heatmaps show region-by-year registered prevalence per 100,000 for ADHD (A), ASD (B), intellectual disability (C), and other developmental disorders (D). Darker shading indicates higher prevalence; the color scale is consistent across panels. This visualization highlights temporal discontinuities and persistent geographic gradients in registry-based prevalence across regions.**

**Supplementary Figure S2. Overall registered prevalence trends of neurodevelopmental disorders among children aged ≤15 years, Kazakhstan, 2014–2024. Single-panel time series comparing registered prevalence per 100,000 children aged ≤15 years across ADHD, ASD, intellectual disability, and other developmental disorders. Lines enable direct comparison of between-disorder trajectories over the study period.**

**Supplementary Figure S3. Registry volume over time: total registered cases and first-time diagnoses per year, 2014–2024. Annual counts of (i) total registered cases in the registry and (ii) first-time diagnoses (first record) plotted against time. The figure contextualises prevalence trends by showing changes in registry throughput and accumulation over the study period.**

**Supplementary Figure S4. Population denominator used for prevalence estimates: total population aged ≤15 years in Kazakhstan, 2014–2024. Annual population counts for children aged ≤15 years were used as denominators for calculating registry-based prevalence per 100,000.**

**Supplementary Figure S5. Changes in diagnostic composition of registered neurodevelopmental disorders by regional grouping, 2014–2024 (children aged ≤15 years) Stacked area plots show the annual proportion of diagnoses attributed to childhood autism (F84.0), atypical autism (F84.1), intellectual disability, and other developmental disorders within selected regional groupings: Astana City (A), Almaty City (B), East Kazakhstan + Abai (C), and all other regions (D). This figure describes how the mix of diagnostic categories contributing to registry counts shifts over time.**

**Supplementary Figure S6. Regional co-trending of autism spectrum disorder subdiagnoses and other neurodevelopmental diagnoses, 2014–2024 (children aged ≤15 years) Cells show the median Spearman rank correlation coefficient (ρ) across regions between annual counts of each ICD-10 ASD subdiagnosis (columns; F84.x) and other neurodevelopmental diagnoses (rows; F80–F89 and tic disorders F95). Correlations were computed within each region across years with sufficient overlap and variability and then summarised across regions using the median ρ and median p-value. Only associations meeting p<0.05 (based on the median p across regions) are shaded; non-significant pairs are marked with “×” to reduce visual noise and limit over-interpretation of unstable estimates. Darker shading indicates positive correlations and lighter shading indicates negative correlations.**

**Registry-based prevalence and segmented-regression fit**

**Supplementary Figure S7. National registry-based prevalence per 100,000 children aged ≤15 years by diagnostic group, Kazakhstan 2014–2024 (solid line, observed; dashed line, segmented-regression fit). Dotted and dashed vertical lines mark the 2019 PMPC restructuring and the 2021 ASD policy expansion; per-panel labels show period-specific annual percent change (2014–2019 | 2019–2021 | 2021–2024). Monochrome, consistent with the main-text figures.**

1.2 **Supplementary Tables**

**Supplementary Table S1. ICD-10–level distribution and demographic profile of the most frequent primary diagnoses among children aged ≤15 years. For each listed ICD-10 primary diagnosis, the table reports diagnostic group, number and percentage of the total sample, age at diagnosis and assessment (median [IQR]), year of diagnosis (median [IQR]), sex distribution, and the most frequent diagnosing specialist, region, socioeconomic status, and ethnicity, alongside citizenship and urban residence percentages.**

| **Primary diagnosis (ICD-10)** | **Diagnostic group** | **N (children)** | **% of total sample** | **Age at diagnosis, years (median [IQR])** | **Year of diagnosis (median [IQR])** | **Age at assessment, years (median [IQR])** | **Male, %** | **Most frequent diagnosing specialist (share, %)** | **Most frequent region (share, %)** | **Most frequent socioeconomic status (share, %)** | **Most frequent ethnicity (share, %)** | **Kazakhstan citizenship, %** | **Urban residence, %** |
| --- | --- | --- | --- | --- | --- | --- | --- | --- | --- | --- | --- | --- | --- |
| Mild intellectual disability, without behavioral impairment (F70.0) | Intellectual disability (ID) | 28704 | 13.36 | 8.2 (6.2–10.4) | 2016 (2013–2019) | 11.0 (9.0–14.0) | 58.9 | Psychiatrist (36.2%) | Almaty City (12.0%) | Out-of-home care (52.1%) | Kazakh (67.8%) | 87.1 | 45.9 |
| Childhood autism (F84.0) | Autism spectrum disorder (ASD) | 27053 | 12.59 | 5.5 (4.4–7.2) | 2020 (2019–2022) | 7.0 (5.0–10.0) | 78.2 | Psychiatrist (56.7%) | Astana City (29.1%) | Home care (40.1%) | Kazakh (75.1%) | 89.2 | 48.1 |
| Moderate intellectual disability, without behavioral impairment (F71.0) | Intellectual disability (ID) | 22924 | 10.67 | 7.0 (5.3–9.4) | 2016 (2012–2019) | 11.0 (8.0–13.0) | 58.1 | Psychiatrist (34.8%) | Atyrau (11.6%) | Home care (43.5%) | Kazakh (72.2%) | 88.5 | 49.7 |
| Mild intellectual disability, with behavioral impairment (F70.1) | Intellectual disability (ID) | 21561 | 10.03 | 8.2 (6.4–10.5) | 2018 (2014–2021) | 11.0 (9.0–13.0) | 66.5 | Psychiatrist (44.8%) | Almaty Region (15.2%) | Out-of-home care (46.8%) | Kazakh (67.6%) | 87.6 | 38.6 |
| Mixed specific developmental disorders (F83) | Other developmental disorders | 17441 | 8.12 | 6.5 (4.5–8.3) | 2017 (2014–2019) | 9.0 (7.0–11.0) | 67.6 | General practitioner / family physician (31.3%) | East Kazakhstan (22.5%) | Out-of-home care (43.7%) | Kazakh (52.6%) | 84.4 | 57.7 |
| Moderate intellectual disability, with behavioral impairment (F71.1) | Intellectual disability (ID) | 17360 | 8.08 | 7.0 (5.4–9.4) | 2018 (2013–2021) | 11.0 (8.0–13.0) | 64.9 | Psychiatrist (50.9%) | Turkistan (13.0%) | Home care (45.5%) | Kazakh (72.0%) | 90 | 55.6 |
| Atypical autism (F84.1) | Autism spectrum disorder (ASD) | 15918 | 7.41 | 6.3 (5.0–7.9) | 2020 (2018–2022) | 8.0 (6.0–10.0) | 78.3 | Psychiatrist (43.5%) | Almaty City (24.6%) | Home care (41.8%) | Kazakh (69.8%) | 83.9 | 38.8 |
| Severe intellectual disability, without behavioral impairment (F72.0) | Intellectual disability (ID) | 13758 | 6.4 | 6.6 (4.7–9.2) | 2016 (2012–2019) | 11.0 (8.0–13.0) | 58.9 | Psychiatrist (33.0%) | Astana City (13.7%) | Home care (66.4%) | Kazakh (73.9%) | 88.2 | 55.4 |
| Phonological disorder (F80.0) | Other developmental disorders | 11206 | 5.21 | 5.9 (4.5–7.5) | 2016 (2014–2019) | 9.0 (6.0–11.0) | 66.7 | General practitioner / family physician (35.4%) | Turkistan (64.5%) | Out-of-home care (48.1%) | Kazakh (66.9%) | 93.2 | 82.6 |
| Severe intellectual disability, with behavioral impairment (F72.1) | Intellectual disability (ID) | 6281 | 2.92 | 7.0 (5.1–10.1) | 2017 (2012–2020) | 11.0 (9.0–13.0) | 63.2 | Psychiatrist (46.1%) | Turkistan (12.8%) | Home care (64.7%) | Kazakh (72.5%) | 90.1 | 53 |
| Mild intellectual disability, other specified (F70.8) | Intellectual disability (ID) | 6228 | 2.9 | 8.3 (6.6–10.5) | 2019 (2015–2021) | 11.0 (8.0–13.0) | 65.2 | Psychiatrist (49.6%) | Astana City (43.9%) | Out-of-home care (38.9%) | Kazakh (73.5%) | 95.2 | 29.6 |
| Moderate intellectual disability, other specified (F71.8) | Intellectual disability (ID) | 4703 | 2.19 | 7.8 (5.8–10.2) | 2019 (2014–2021) | 11.0 (8.0–13.0) | 62 | Psychiatrist (46.7%) | Astana City (34.5%) | Home care (40.5%) | Kazakh (76.1%) | 95.3 | 46.5 |
| Severe intellectual disability, other specified (F72.8) | Intellectual disability (ID) | 2427 | 1.13 | 7.3 (5.5–10.2) | 2018 (2014–2020) | 11.0 (8.0–13.0) | 61.2 | Psychiatrist (41.9%) | Karagandy (26.0%) | Home care (61.0%) | Kazakh (73.5%) | 93.1 | 47.3 |
| Profound intellectual disability, without behavioral impairment (F73.0) | Intellectual disability (ID) | 2312 | 1.08 | 6.3 (4.3–8.6) | 2015 (2011–2019) | 11.0 (8.0–13.0) | 58.1 | Psychiatrist (28.8%) | Almaty City (13.5%) | Home care (62.4%) | Kazakh (69.4%) | 85.8 | 51.3 |
| Other speech and language disorders (F80.8) | Other developmental disorders | 1715 | 0.8 | 5.7 (4.3–7.1) | 2014 (2012–2017) | 9.0 (7.0–11.0) | 63.9 | Other physician (37.5%) | Karagandy (39.5%) | Out-of-home care (55.5%) | Kazakh (57.3%) | 83 | 62.7 |
| Mixed disorder of scholastic skills (F81.3) | Other developmental disorders | 1559 | 0.73 | 8.8 (7.5–10.4) | 2016 (2013–2019) | 11.0 (10.0–13.0) | 65.7 | Other physician (29.3%) | Akmola (29.1%) | Out-of-home care (64.8%) | Kazakh (48.9%) | 77.7 | 51.6 |
| Mild intellectual disability, unspecified (F70.9) | Intellectual disability (ID) | 1540 | 0.72 | 8.1 (6.4–10.1) | 2014 (2011–2017) | 12.0 (10.0–14.0) | 53.9 | Psychiatrist (39.6%) | Karagandy (40.1%) | Out-of-home care (67.9%) | Kazakh (59.2%) | 86.2 | 35.8 |
| Specific reading disorder (dyslexia) (F81.0) | Other developmental disorders | 1321 | 0.61 | 8.0 (6.9–9.7) | 2017 (2014–2019) | 11.0 (9.0–12.0) | 65.6 | General practitioner / family physician (40.8%) | Turkistan (49.0%) | Out-of-home care (65.9%) | Kazakh (57.8%) | 88.9 | 67.8 |
| ADHD, predominantly inattentive (F90.0) | Attention-deficit/hyperactivity disorder (ADHD) | 1208 | 0.56 | 6.9 (5.5–8.6) | 2015 (2013–2019) | 10.0 (8.0–12.0) | 78.5 | General practitioner / family physician (44.3%) | Turkistan (57.8%) | Out-of-home care (58.7%) | Kazakh (46.4%) | 89.2 | 69.4 |
| ADHD, predominantly hyperactive/impulsive (F90.1) | Attention-deficit/hyperactivity disorder (ADHD) | 1175 | 0.55 | 7.3 (6.0–8.9) | 2016 (2013–2019) | 10.0 (8.0–12.0) | 84.7 | General practitioner / family physician (31.8%) | Kostanay (31.3%) | Out-of-home care (62.7%) | Russian (47.1%) | 70.2 | 55.8 |
| Overactive disorder with intellectual disability (F84.4) | Autism spectrum disorder (ASD) | 870 | 0.4 | 5.3 (4.3–7.2) | 2017 (2013–2020) | 9.0 (7.0–12.0) | 77.8 | Psychiatrist (42.6%) | Turkistan (52.0%) | Out-of-home care (47.1%) | Kazakh (63.9%) | 91.4 | 54.3 |
| Expressive language disorder (F80.1) | Other developmental disorders | 849 | 0.4 | 6.1 (4.7–7.8) | 2018 (2014–2018) | 9.0 (7.0–11.0) | 69.5 | Psychiatrist (40.5%) | Turkistan (53.7%) | Out-of-home care (51.2%) | Kazakh (60.7%) | 91.5 | 70.1 |
| Profound intellectual disability, with behavioral impairment (F73.1) | Intellectual disability (ID) | 784 | 0.36 | 5.9 (4.3–8.9) | 2013 (2011–2018) | 11.0 (9.0–13.0) | 64.8 | General practitioner / family physician (34.8%) | Almaty Region (26.0%) | Home care (67.7%) | Kazakh (74.7%) | 82.7 | 43.4 |
| Other scholastic skill disorders (F81.8) | Other developmental disorders | 731 | 0.34 | 8.0 (7.3–9.1) | 2016 (2014–2019) | 11.0 (9.0–13.0) | 67.9 | Psychiatrist (51.8%) | Karagandy (71.8%) | Out-of-home care (65.5%) | Kazakh (39.6%) | 85.5 | 78.5 |
| Moderate intellectual disability, unspecified (F71.9) | Intellectual disability (ID) | 608 | 0.28 | 7.5 (5.4–9.5) | 2015 (2013–2018) | 11.0 (9.0–13.2) | 58.6 | Psychiatrist (34.4%) | Karagandy (41.0%) | Out-of-home care (43.5%) | Kazakh (64.8%) | 85 | 52.8 |
| Asperger syndrome (F84.5) | Autism spectrum disorder (ASD) | 554 | 0.26 | 8.2 (6.2–10.2) | 2020 (2017–2022) | 11.0 (8.0–13.0) | 88.6 | Psychiatrist (51.8%) | Astana City (43.0%) | Out-of-home care (38.0%) | Kazakh (67.2%) | 91.2 | 43.7 |
| Other disorders of psychological development (F88) | Other developmental disorders | 550 | 0.26 | 6.4 (4.6–8.1) | 2012 (2010–2017) | 11.0 (8.0–13.0) | 71.6 | Other physician (63.8%) | Karagandy (91.8%) | Out-of-home care (69.3%) | Russian (35.7%) | 63.3 | 68.4 |
| Scholastic disorder, unspecified (F81.9) | Other developmental disorders | 499 | 0.23 | 8.2 (7.4–9.3) | 2015 (2013–2017) | 11.0 (9.0–13.0) | 70.5 | Psychiatrist (37.1%) | Karagandy (58.7%) | Out-of-home care (63.0%) | Kazakh (35.5%) | 74.9 | 68.9 |
| Other intellectual disability, without behavioral impairment (F78.0) | Intellectual disability (ID) | 360 | 0.17 | 4.5 (3.1–7.8) | 2014 (2010–2019) | 9.0 (6.0–12.0) | 46.4 | Psychiatrist (30.8%) | Almaty Region (43.3%) | Home care (60.1%) | Kazakh (64.4%) | 82.8 | 49.4 |
| Profound intellectual disability, other specified (F73.8) | Intellectual disability (ID) | 340 | 0.16 | 6.4 (4.4–8.4) | 2016 (2012–2018) | 10.0 (7.0–12.0) | 50.9 | Other physician (42.1%) | Mangystau (29.7%) | Home care (73.3%) | Kazakh (74.9%) | 92.6 | 58.5 |
| Severe intellectual disability, unspecified (F72.9) | Intellectual disability (ID) | 328 | 0.15 | 6.6 (4.8–9.8) | 2015 (2010–2019) | 12.0 (9.0–14.0) | 34.1 | Psychiatrist (41.8%) | Karagandy (43.3%) | Home care (65.3%) | Kazakh (57.5%) | 94.8 | 59.8 |
| Other pervasive developmental disorders (F84.8) | Autism spectrum disorder (ASD) | 321 | 0.15 | 5.9 (4.7–8.4) | 2017 (2014–2019) | 9.0 (6.0–12.0) | 72.3 | Psychiatrist (52.6%) | West Kazakhstan (28.7%) | Out-of-home care (48.0%) | Kazakh (53.3%) | 88.8 | 50.5 |
| Speech and language disorder, unspecified (F80.9) | Other developmental disorders | 230 | 0.11 | 5.1 (4.1–6.4) | 2014 (2012–2018) | 8.0 (6.0–10.0) | 63.5 | Other physician (35.2%) | Karagandy (24.3%) | Out-of-home care (50.5%) | Russian (46.0%) | 87.4 | 51.7 |
| Profound intellectual disability, unspecified (F73.9) | Intellectual disability (ID) | 202 | 0.09 | 6.6 (4.6–10.3) | 2013 (2012–2018) | 11.0 (9.0–13.0) | 54 | General practitioner / family physician (27.2%) | Karagandy (44.6%) | Home care (74.8%) | Kazakh (66.5%) | 79.7 | 65.3 |
| Transient tic disorder (F95.0) | Other developmental disorders | 168 | 0.08 | 8.5 (7.4–9.8) | 2015 (2012–2017) | 11.0 (9.8–13.0) | 78.6 | General practitioner / family physician (30.4%) | East Kazakhstan (32.7%) | Out-of-home care (81.2%) | Kazakh (48.7%) | 75.6 | 76.8 |
| Pervasive developmental disorder, unspecified (F84.9) | Autism spectrum disorder (ASD) | 143 | 0.07 | 5.5 (4.4–7.0) | 2017 (2013–2020) | 7.0 (6.0–10.0) | 83.9 | Psychiatrist (48.3%) | Pavlodar (37.1%) | School-age student (44.9%) | Kazakh (59.8%) | 92.3 | 53.1 |
| Receptive language disorder (F80.2) | Other developmental disorders | 113 | 0.05 | 6.5 (5.4–8.5) | 2015 (2014–2017) | 10.0 (8.0–12.0) | 54 | Psychiatrist (38.1%) | Turkistan (60.2%) | Out-of-home care (62.4%) | Kazakh (61.5%) | 100 | 76.1 |
| Rett syndrome (F84.2) | Autism spectrum disorder (ASD) | 100 | 0.05 | 6.0 (4.0–8.6) | 2019 (2015–2021) | 8.0 (6.0–11.0) | 19 | Psychiatrist (50.0%) | Aktobe (24.0%) | Home care (80.0%) | Kazakh (89.1%) | 91 | 59 |
| Chronic motor or vocal tic disorder (F95.1) | Other developmental disorders | 74 | 0.03 | 8.7 (7.5–10.9) | 2015 (2014–2017) | 12.0 (10.0–13.0) | 55.4 | Public health physician (54.1%) | North Kazakhstan (51.4%) | Out-of-home care (58.3%) | Russian (65.0%) | 66.2 | 52.7 |
| Other intellectual disability, with behavioral impairment (F78.1) | Intellectual disability (ID) | 67 | 0.03 | 4.8 (4.6–6.8) | 2015 (2013–2016) | 9.0 (7.0–12.0) | 44.8 | Other physician (50.7%) | Mangystau (25.4%) | Home care (70.4%) | Kazakh (51.3%) | 88.1 | 23.9 |
| Moderate intellectual disability (F71) | Intellectual disability (ID) | 65 | 0.03 | 6.7 (5.0–9.4) | 2011 (2010–2013) | 12.0 (10.0–14.0) | 53.8 | General practitioner / family physician (78.5%) | Mangystau (80.0%) | Out-of-home care (63.2%) | Kazakh (84.4%) | 58.5 | 1.5 |
| Specific spelling disorder (F81.1) | Other developmental disorders | 59 | 0.03 | 8.7 (7.0–10.4) | 2019 (2017–2021) | 10.0 (9.0–13.0) | 78 | General practitioner / family physician (37.3%) | Shymkent City (33.9%) | Out-of-home care (45.5%) | Kazakh (70.8%) | 64.4 | 30.5 |
| Unspecified disorder of psychological development (F89) | Other developmental disorders | 57 | 0.03 | 6.8 (4.8–7.7) | 2013 (2012–2020) | 11.0 (8.0–13.0) | 52.6 | Other physician (38.6%) | Almaty Region (35.1%) | Out-of-home care (89.2%) | Kazakh (71.1%) | 77.2 | 29.8 |
| Mild intellectual disability (F70) | Intellectual disability (ID) | 55 | 0.03 | 7.4 (5.4–8.9) | 2012 (2010–2012) | 12.0 (9.5–14.0) | 61.8 | Nurse / mid-level practitioner (32.7%) | Almaty Region (32.7%) | Out-of-home care (68.3%) | Kazakh (69.6%) | 65.5 | 20 |
| Tourette syndrome (F95.2) | Other developmental disorders | 51 | 0.02 | 10.3 (9.6–11.6) | 2016 (2013–2020) | 12.0 (11.0–14.0) | 62.7 | Public health physician (43.1%) | Astana City (27.5%) | School-age student (46.2%) | Kazakh (44.7%) | 84.3 | 51 |
| Other childhood disintegrative disorder (F84.3) | Autism spectrum disorder (ASD) | 48 | 0.02 | 6.7 (4.7–8.1) | 2015 (2013–2017) | 8.0 (6.0–11.0) | 89.6 | General practitioner / family physician (29.2%) | East Kazakhstan (35.4%) | Out-of-home care (57.8%) | Kazakh (75.0%) | 91.7 | 72.9 |
| Unspecified intellectual disability, without behavioral impairment (F79.0) | Intellectual disability (ID) | 46 | 0.02 | 7.5 (6.8–8.8) | 2019 (2017–2019) | 10.0 (7.0–12.8) | 47.8 | Psychiatrist (39.1%) | Zhambyl (73.9%) | Home care (76.9%) | Kazakh (46.4%) | 100 | 76.1 |
| Severe intellectual disability (F72) | Intellectual disability (ID) | 45 | 0.02 | 6.1 (3.6–6.7) | 2013 (2010–2013) | 9.0 (7.0–11.0) | 57.8 | General practitioner / family physician (64.4%) | Mangystau (66.7%) | Home care (52.2%) | Kazakh (97.5%) | 66.7 | 2.2 |
| Specific developmental disorder of motor function (F82) | Other developmental disorders | 34 | 0.02 | 5.8 (5.5–6.6) | 2013 (2012–2014) | 9.0 (8.0–11.0) | 32.4 | Other physician (32.4%) | Turkistan (32.4%) | Out-of-home care (64.0%) | Kazakh (44.8%) | 100 | 41.2 |
| Acquired aphasia with epilepsy (F80.3) | Other developmental disorders | 27 | 0.01 | 6.1 (4.0–7.2) | 2012 (2011–2012) | 10.0 (9.0–11.0) | 59.3 | General practitioner / family physician (55.6%) | Turkistan (92.6%) | Out-of-home care (47.6%) | Uzbek (44.0%) | 100 | 92.6 |
| Other ADHD (F90.8) | Attention-deficit/hyperactivity disorder (ADHD) | 23 | 0.01 | 6.8 (3.3–9.9) | 2015 (2012–2019) | 9.0 (7.0–12.0) | 91.3 | Other physician (73.9%) | Akmola (43.5%) | Out-of-home care (100.0%) | Ukrainian (100.0%) | 100 | 8.7 |
| Unspecified intellectual disability, unspecified (F79.9) | Intellectual disability (ID) | 13 | 0.01 | 3.2 (3.2–7.5) | 2010 (2010–2017) | 11.0 (9.0–12.0) | 100 | Psychiatrist (53.8%) | Pavlodar (53.8%) | Home care (100.0%) | Russian (53.8%) | 100 | 53.8 |
| Unspecified intellectual disability, with behavioral impairment (F79.1) | Intellectual disability (ID) | 13 | 0.01 | 5.7 (5.7–10.2) | 2014 (2013–2019) | 10.0 (8.0–12.0) | 61.5 | Psychiatrist (46.2%) | Karagandy (46.2%) | Out-of-home care (71.4%) | Russian (54.5%) | 100 | 84.6 |
| Other intellectual disability, other specified (F78.8) | Intellectual disability (ID) | 12 | 0.01 | 3.3 (3.3–11.7) | 2012 (2012–2021) | 9.5 (6.8–12.2) | 8.3 | Nurse / mid-level practitioner (58.3%) | Jetisu (75.0%) | Home care (100.0%) | Kazakh (100.0%) | 100 | 58.3 |
| Specific developmental disorders of speech and language (F80) | Other developmental disorders | 12 | 0.01 | 4.8 (4.2–5.9) | 2009 (2008–2011) | 9.5 (7.0–12.5) | 58.3 | General practitioner / family physician (58.3%) | Mangystau (50.0%) | Out-of-home care (100.0%) | Kazakh (81.8%) | 58.3 | 41.7 |
| Specific developmental disorders of scholastic skills (F81) | Other developmental disorders | 11 | 0.01 | 7.8 (7.3–7.9) | 2012 (2011–2012) | 10.0 (9.0–11.0) | 63.6 | Public health physician (100.0%) | Karagandy (100.0%) | Out-of-home care (100.0%) | Russian (50.0%) | 18.2 | 100 |
| Specific disorder of arithmetical skills (dyscalculia) (F81.2) | Other developmental disorders | 10 | 0 | 9.2 (9.2–10.6) | 2018 (2015–2018) | 12.5 (11.2–13.8) | 100 | Psychiatrist (60.0%) | Akmola (60.0%) | School-age student (100.0%) | Kazakh (60.0%) | 100 | 40 |
| Tic disorder, unspecified (F95.9) | Other developmental disorders | 8 | 0 | 9.2 (9.2–11.0) | 2015 (2015–2016) | 11.5 (10.8–12.2) | 75 | Public health physician (87.5%) | Karagandy (87.5%) | School-age student (62.5%) | Kazakh (87.5%) | 100 | 87.5 |
| ADHD, unspecified (F90.9) | Attention-deficit/hyperactivity disorder (ADHD) | 7 | 0 | 10.5 (9.3–10.5) | 2019 (2019–2021) | 12.0 (9.5–13.5) | 100 | General practitioner / family physician (100.0%) | Shymkent City (100.0%) | School-age student (71.4%) | NA | 100 | 0 |
| Profound intellectual disability (F73) | Intellectual disability (ID) | 7 | 0 | 1.6 (1.5–3.3) | 2009 (2009–2010) | 7.0 (5.5–9.0) | 57.1 | Unknown (42.9%) | Almaty City (100.0%) | Out-of-home care (66.7%) | Russian (40.0%) | 71.4 | 0 |
| Other tic disorders (F95.8) | Other developmental disorders | 7 | 0 | 6.5 (4.5–11.6) | 2015 (2007–2016) | 13.0 (10.5–14.0) | 85.7 | Public health physician (57.1%) | Kostanay (71.4%) | Out-of-home care (85.7%) | Russian (50.0%) | 57.1 | 28.6 |
| Other intellectual disability, unspecified (F78.9) | Intellectual disability (ID) | 6 | 0 | 10.0 (8.6–12.1) | 2020 (2019–2022) | 10.5 (9.2–11.8) | 0 | Other physician (66.7%) | Mangystau (100.0%) | Home care (100.0%) | Kazakh (100.0%) | 100 | 33.3 |
| Pervasive developmental disorders (F84) | Autism spectrum disorder (ASD) | 3 | 0 | 10.2 (10.2–10.2) | 2013 (2013–2013) | 13.0 (12.5–13.5) | 100 | General practitioner / family physician (100.0%) | Mangystau (100.0%) | Home care (100.0%) | Kazakh (100.0%) | 100 | 0 |

**Supplementary Table S2. Complete ICD-10 code inventory by diagnostic group**

Every primary ICD-10 code observed in the analytic cohort (children aged ≤15 years, 2014–2024 pooled records), with record counts and within-group percentages.

**ASD (F84.*) — n = 45,010; 9 distinct codes**

| **ICD-10 code** | **Records (n)** | **% within group** |
| --- | --- | --- |
| F84.0 | 27,053 | 60.10 |
| F84.1 | 15,918 | 35.37 |
| F84.4 | 870 | 1.93 |
| F84.5 | 554 | 1.23 |
| F84.8 | 321 | 0.71 |
| F84.9 | 143 | 0.32 |
| F84.2 | 100 | 0.22 |
| F84.3 | 48 | 0.11 |
| F84 | 3 | 0.01 |

**ADHD (F90.*) — n = 2,413; 4 distinct codes**

| **ICD-10 code** | **Records (n)** | **% within group** |
| --- | --- | --- |
| F90.0 | 1,208 | 50.06 |
| F90.1 | 1,175 | 48.69 |
| F90.8 | 23 | 0.95 |
| F90.9 | 7 | 0.29 |

**Intellectual disability (F70–F79) — n = 130,749; 27 distinct codes**

| **ICD-10 code** | **Records (n)** | **% within group** |
| --- | --- | --- |
| F70.0 | 28,704 | 21.95 |
| F71.0 | 22,924 | 17.53 |
| F70.1 | 21,561 | 16.49 |
| F71.1 | 17,360 | 13.28 |
| F72.0 | 13,758 | 10.52 |
| F72.1 | 6,281 | 4.80 |
| F70.8 | 6,228 | 4.76 |
| F71.8 | 4,703 | 3.60 |
| F72.8 | 2,427 | 1.86 |
| F73.0 | 2,312 | 1.77 |
| F70.9 | 1,540 | 1.18 |
| F73.1 | 784 | 0.60 |
| F71.9 | 608 | 0.47 |
| F78.0 | 360 | 0.28 |
| F73.8 | 340 | 0.26 |
| F72.9 | 328 | 0.25 |
| F73.9 | 202 | 0.15 |
| F78.1 | 67 | 0.05 |
| F71 | 65 | 0.05 |
| F70 | 55 | 0.04 |
| F79.0 | 46 | 0.04 |
| F72 | 45 | 0.03 |
| F79.1 | 13 | 0.01 |
| F79.9 | 13 | 0.01 |
| F78.8 | 12 | 0.01 |
| F73 | 7 | 0.01 |
| F78.9 | 6 | 0.00 |

**Other developmental disorders (F80–F89, F95) — n = 36,732; 23 distinct codes**

| **ICD-10 code** | **Records (n)** | **% within group** |
| --- | --- | --- |
| F83 | 17,441 | 47.48 |
| F80.0 | 11,206 | 30.51 |
| F80.8 | 1,715 | 4.67 |
| F81.3 | 1,559 | 4.24 |
| F81.0 | 1,321 | 3.60 |
| F80.1 | 849 | 2.31 |
| F81.8 | 731 | 1.99 |
| F88 | 550 | 1.50 |
| F81.9 | 499 | 1.36 |
| F80.9 | 230 | 0.63 |
| F95.0 | 168 | 0.46 |
| F80.2 | 113 | 0.31 |
| F95.1 | 74 | 0.20 |
| F81.1 | 59 | 0.16 |
| F89 | 57 | 0.16 |
| F95.2 | 51 | 0.14 |
| F82 | 34 | 0.09 |
| F80.3 | 27 | 0.07 |
| F80 | 12 | 0.03 |
| F81 | 11 | 0.03 |
| F81.2 | 10 | 0.03 |
| F95.9 | 8 | 0.02 |
| F95.8 | 7 | 0.02 |

**Supplementary Table S2b. Composition model results: associations between demographic, socioeconomic, and specialist composition and registry-based prevalence across disorder groups. Model estimates (odds ratios) for predictors defined at the unit-of-analysis level (e.g., region-year or registry strata), including registry year, demographic composition measures, socioeconomic composition, and diagnosing specialist composition. Results are reported for ADHD, ASD, intellectual disability, and other developmental disorders with OR and 95% credible intervals, alongside MCMC diagnostics.**

| **Disorder** | **term** | **Predictor** | **OR** | **OR_low** | **OR_high** | **ess_bulk** | **r_hat** | **OR (95% CrI)** |
| --- | --- | --- | --- | --- | --- | --- | --- | --- |
| ADHD | year_c | Year (per 1-year increase) | 1.066092399 | 0.962712941 | 1.188866105 | 1977 | 1 | 1.07 (0.96, 1.19) |
| ADHD | prop_male_c10 | Male composition (+10pp) | 1.081122659 | 0.703280122 | 1.741940985 | 3491 | 1 | 1.08 (0.70, 1.74) |
| ADHD | age_mean_c | Mean age at diagnosis (centered, +1 year) | 1.025315121 | 0.881614847 | 1.199614194 | 2052 | 1 | 1.03 (0.88, 1.20) |
| ADHD | prop_non_kz_c10 | Non-citizen composition (+10pp) | 1.138828383 | 0.93332668 | 1.373002572 | 2972 | 1 | 1.14 (0.93, 1.37) |
| ADHD | prop_city_c10 | Urban composition (+10pp) | 1.021222052 | 0.965605416 | 1.078962574 | 2036 | 1 | 1.02 (0.97, 1.08) |
| ADHD | eth_share_central_asian_c10 | Ethnicity: Central Asian (+10pp) | 1.217744041 | 1.027367803 | 1.43046562 | 1903 | 1 | 1.22 (1.03, 1.43) |
| ADHD | eth_share_slavic_c10 | Ethnicity: Slavic (+10pp) | 1.200814408 | 1 | 1.437635859 | 1958 | 1 | 1.20 (1.00, 1.44) |
| ADHD | ses_share_home_care_c10 | SES: Home Care (+10pp) | 0.826132588 | 0.717487323 | 0.963676135 | 3034 | 1 | 0.83 (0.72, 0.96) |
| ADHD | ses_share_out_of_home_care_c10 | SES: Out Of Home Care (+10pp) | 1.308655141 | 1.05865581 | 1.576173383 | 2246 | 1 | 1.31 (1.06, 1.58) |
| ADHD | ses_share_preschool_c10 | SES: Preschool (+10pp) | 1.122995872 | 0.784271514 | 1.640498239 | 2853 | 1 | 1.12 (0.78, 1.64) |
| ADHD | ses_share_school_age_student_c10 | SES: School Age Student (+10pp) | 0.894938749 | 0.723250242 | 1.1491241 | 2639 | 1 | 0.89 (0.72, 1.15) |
| ADHD | spec_share_gp_family_c10 | Specialist: Gp Family (+10pp) | 1.214096283 | 0.955997482 | 1.571451949 | 1350 | 1 | 1.21 (0.96, 1.57) |
| ADHD | spec_share_neurologist_c10 | Specialist: Neurologist (+10pp) | 0.939882887 | 0.549910358 | 1.612845483 | 4540 | 1 | 0.94 (0.55, 1.61) |
| ADHD | spec_share_nurse_midlevel_c10 | Specialist: Nurse Midlevel (+10pp) | 0.883379841 | 0.669650061 | 1.169995614 | 1635 | 1 | 0.88 (0.67, 1.17) |
| ADHD | spec_share_other_physician_c10 | Specialist: Other Physician (+10pp) | 0.971416464 | 0.771823023 | 1.247323431 | 1444 | 1 | 0.97 (0.77, 1.25) |
| ADHD | spec_share_pediatrician_c10 | Specialist: Pediatrician (+10pp) | 1.186490749 | 0.853849782 | 1.574597997 | 1623 | 1 | 1.19 (0.85, 1.57) |
| ADHD | spec_share_psychiatrist_c10 | Specialist: Psychiatrist (+10pp) | 1 | 0.778800783 | 1.253575665 | 1413 | 1 | 1.00 (0.78, 1.25) |
| ADHD | spec_share_psychologist_c10 | Specialist: Psychologist (+10pp) | 0.991040379 | 0.563267855 | 1.726333853 | 4492 | 1 | 0.99 (0.56, 1.73) |
| ADHD | spec_share_public_health_c10 | Specialist: Public Health (+10pp) | 1.049170655 | 0.822834658 | 1.353914464 | 1377 | 1 | 1.05 (0.82, 1.35) |
| ASD | year_c | Year (per 1-year increase) | 1.280179113 | 1.249820574 | 1.31127507 | 2604 | 1 | 1.28 (1.25, 1.31) |
| ASD | prop_male_c10 | Male composition (+10pp) | 1.738460584 | 1.518920674 | 1.969933922 | 3724 | 1 | 1.74 (1.52, 1.97) |
| ASD | age_mean_c | Mean age at diagnosis (centered, +1 year) | 1.025315121 | 0.992031915 | 1.05865581 | 2299 | 1 | 1.03 (0.99, 1.06) |
| ASD | prop_non_kz_c10 | Non-citizen composition (+10pp) | 0.861569115 | 0.815462371 | 0.9111935 | 2286 | 1 | 0.86 (0.82, 0.91) |
| ASD | prop_city_c10 | Urban composition (+10pp) | 1.028395684 | 1.016128685 | 1.040810774 | 2870 | 1 | 1.03 (1.02, 1.04) |
| ASD | eth_share_central_asian_c10 | Ethnicity: Central Asian (+10pp) | 1.062898914 | 1.017145322 | 1.11071061 | 2260 | 1 | 1.06 (1.02, 1.11) |
| ASD | eth_share_slavic_c10 | Ethnicity: Slavic (+10pp) | 0.994017964 | 0.937067463 | 1.044982355 | 2374 | 1 | 0.99 (0.94, 1.04) |
| ASD | ses_share_home_care_c10 | SES: Home Care (+10pp) | 1.14797555 | 1.105170918 | 1.192438059 | 2846 | 1 | 1.15 (1.11, 1.19) |
| ASD | ses_share_out_of_home_care_c10 | SES: Out Of Home Care (+10pp) | 1.021222052 | 0.989060279 | 1.052322893 | 3454 | 1 | 1.02 (0.99, 1.05) |
| ASD | ses_share_preschool_c10 | SES: Preschool (+10pp) | 1.081122659 | 1.033550539 | 1.130884421 | 2286 | 1 | 1.08 (1.03, 1.13) |
| ASD | ses_share_school_age_student_c10 | SES: School Age Student (+10pp) | 0.844508903 | 0.799315134 | 0.888696053 | 2648 | 1 | 0.84 (0.80, 0.89) |
| ASD | spec_share_gp_family_c10 | Specialist: Gp Family (+10pp) | 0.852996359 | 0.800114849 | 0.91210515 | 1252 | 1 | 0.85 (0.80, 0.91) |
| ASD | spec_share_neurologist_c10 | Specialist: Neurologist (+10pp) | 0.617547062 | 0.478547406 | 0.79612426 | 3103 | 1 | 0.62 (0.48, 0.80) |
| ASD | spec_share_nurse_midlevel_c10 | Specialist: Nurse Midlevel (+10pp) | 1.042894479 | 0.955041962 | 1.125244111 | 1802 | 1 | 1.04 (0.96, 1.13) |
| ASD | spec_share_other_physician_c10 | Specialist: Other Physician (+10pp) | 0.874590065 | 0.822012235 | 0.926816207 | 1230 | 1 | 0.87 (0.82, 0.93) |
| ASD | spec_share_pediatrician_c10 | Specialist: Pediatrician (+10pp) | 0.923116346 | 0.871970226 | 0.983143685 | 1489 | 1 | 0.92 (0.87, 0.98) |
| ASD | spec_share_psychiatrist_c10 | Specialist: Psychiatrist (+10pp) | 0.907556006 | 0.852143789 | 0.961750709 | 1256 | 1 | 0.91 (0.85, 0.96) |
| ASD | spec_share_psychologist_c10 | Specialist: Psychologist (+10pp) | 2.415726331 | 1.902178865 | 3.028295742 | 3178 | 1 | 2.42 (1.90, 3.03) |
| ASD | spec_share_public_health_c10 | Specialist: Public Health (+10pp) | 0.908464016 | 0.847893704 | 0.968506582 | 1210 | 1 | 0.91 (0.85, 0.97) |
| ID | year_c | Year (per 1-year increase) | 0.972388367 | 0.954087398 | 0.991040379 | 2254 | 1 | 0.97 (0.95, 0.99) |
| ID | prop_male_c10 | Male composition (+10pp) | 0.521002729 | 0.469540839 | 0.575221555 | 2778 | 1 | 0.52 (0.47, 0.58) |
| ID | age_mean_c | Mean age at diagnosis (centered, +1 year) | 1.016128685 | 0.989060279 | 1.042894479 | 2738 | 1 | 1.02 (0.99, 1.04) |
| ID | prop_non_kz_c10 | Non-citizen composition (+10pp) | 0.863293977 | 0.830273595 | 0.900324523 | 1934 | 1 | 0.86 (0.83, 0.90) |
| ID | prop_city_c10 | Urban composition (+10pp) | 1.016128685 | 1.006018036 | 1.026340948 | 2455 | 1 | 1.02 (1.01, 1.03) |
| ID | eth_share_central_asian_c10 | Ethnicity: Central Asian (+10pp) | 1.171166195 | 1.135417018 | 1.206833515 | 2655 | 1 | 1.17 (1.14, 1.21) |
| ID | eth_share_slavic_c10 | Ethnicity: Slavic (+10pp) | 1.082204322 | 1.035619709 | 1.129754102 | 2313 | 1 | 1.08 (1.04, 1.13) |
| ID | ses_share_home_care_c10 | SES: Home Care (+10pp) | 1.119631933 | 1.083287068 | 1.153729802 | 2727 | 1 | 1.12 (1.08, 1.15) |
| ID | ses_share_out_of_home_care_c10 | SES: Out Of Home Care (+10pp) | 0.989060279 | 0.965605416 | 1.016128685 | 3081 | 1 | 0.99 (0.97, 1.02) |
| ID | ses_share_preschool_c10 | SES: Preschool (+10pp) | 0.910282762 | 0.876340995 | 0.945539136 | 2920 | 1 | 0.91 (0.88, 0.95) |
| ID | ses_share_school_age_student_c10 | SES: School Age Student (+10pp) | 0.98412732 | 0.939882887 | 1.029424594 | 2887 | 1 | 0.98 (0.94, 1.03) |
| ID | spec_share_gp_family_c10 | Specialist: Gp Family (+10pp) | 0.856415177 | 0.808156137 | 0.907556006 | 1178 | 1 | 0.86 (0.81, 0.91) |
| ID | spec_share_neurologist_c10 | Specialist: Neurologist (+10pp) | 2.37738228 | 1.948383394 | 2.80948165 | 2781 | 1 | 2.38 (1.95, 2.81) |
| ID | spec_share_nurse_midlevel_c10 | Specialist: Nurse Midlevel (+10pp) | 0.826132588 | 0.772595232 | 0.882496903 | 1251 | 1 | 0.83 (0.77, 0.88) |
| ID | spec_share_other_physician_c10 | Specialist: Other Physician (+10pp) | 1.035619709 | 0.980198673 | 1.094174284 | 1143 | 1 | 1.04 (0.98, 1.09) |
| ID | spec_share_pediatrician_c10 | Specialist: Pediatrician (+10pp) | 1.09089668 | 1.037693021 | 1.154884109 | 1245 | 1 | 1.09 (1.04, 1.15) |
| ID | spec_share_psychiatrist_c10 | Specialist: Psychiatrist (+10pp) | 0.983143685 | 0.934260474 | 1.044982355 | 1120 | 1 | 0.98 (0.93, 1.04) |
| ID | spec_share_psychologist_c10 | Specialist: Psychologist (+10pp) | 5.370924211 | 4.46379812 | 6.410902137 | 3183 | 1 | 5.37 (4.46, 6.41) |
| ID | spec_share_public_health_c10 | Specialist: Public Health (+10pp) | 0.981179362 | 0.924039924 | 1.043937895 | 1121 | 1 | 0.98 (0.92, 1.04) |
| Other NDD | year_c | Year (per 1-year increase) | 0.892257956 | 0.865887748 | 0.915760877 | 1692 | 1 | 0.89 (0.87, 0.92) |
| Other NDD | prop_male_c10 | Male composition (+10pp) | 1.056540615 | 0.914845574 | 1.238622655 | 2700 | 1 | 1.06 (0.91, 1.24) |
| Other NDD | age_mean_c | Mean age at diagnosis (centered, +1 year) | 1.172337947 | 1.117394907 | 1.234912355 | 1976 | 1 | 1.17 (1.12, 1.23) |
| Other NDD | prop_non_kz_c10 | Non-citizen composition (+10pp) | 0.850441205 | 0.802518798 | 0.903029552 | 2804 | 1 | 0.85 (0.80, 0.90) |
| Other NDD | prop_city_c10 | Urban composition (+10pp) | 0.97433509 | 0.955997482 | 0.993024443 | 2463 | 1 | 0.97 (0.96, 0.99) |
| Other NDD | eth_share_central_asian_c10 | Ethnicity: Central Asian (+10pp) | 0.98412732 | 0.943649947 | 1.031485504 | 2257 | 1 | 0.98 (0.94, 1.03) |
| Other NDD | eth_share_slavic_c10 | Ethnicity: Slavic (+10pp) | 1.158353963 | 1.087628894 | 1.23367806 | 2051 | 1 | 1.16 (1.09, 1.23) |
| Other NDD | ses_share_home_care_c10 | SES: Home Care (+10pp) | 1.004008011 | 0.958869781 | 1.051271096 | 2738 | 1 | 1.00 (0.96, 1.05) |
| Other NDD | ses_share_out_of_home_care_c10 | SES: Out Of Home Care (+10pp) | 0.905742708 | 0.859847699 | 0.955997482 | 2263 | 1 | 0.91 (0.86, 0.96) |
| Other NDD | ses_share_preschool_c10 | SES: Preschool (+10pp) | 0.903029552 | 0.817912432 | 1.004008011 | 2795 | 1 | 0.90 (0.82, 1.00) |
| Other NDD | ses_share_school_age_student_c10 | SES: School Age Student (+10pp) | 1.449183073 | 1.341783904 | 1.569881282 | 2263 | 1 | 1.45 (1.34, 1.57) |
| Other NDD | spec_share_gp_family_c10 | Specialist: Gp Family (+10pp) | 1.032517505 | 0.900324523 | 1.167657961 | 930 | 1 | 1.03 (0.90, 1.17) |
| Other NDD | spec_share_neurologist_c10 | Specialist: Neurologist (+10pp) | 2.119118075 | 1.59042297 | 2.787095461 | 2087 | 1 | 2.12 (1.59, 2.79) |
| Other NDD | spec_share_nurse_midlevel_c10 | Specialist: Nurse Midlevel (+10pp) | 0.683861409 | 0.593332695 | 0.777244738 | 1005 | 1 | 0.68 (0.59, 0.78) |
| Other NDD | spec_share_other_physician_c10 | Specialist: Other Physician (+10pp) | 0.822834658 | 0.717487323 | 0.918512284 | 941 | 1 | 0.82 (0.72, 0.92) |
| Other NDD | spec_share_pediatrician_c10 | Specialist: Pediatrician (+10pp) | 0.584499122 | 0.510175752 | 0.662986932 | 1151 | 1 | 0.58 (0.51, 0.66) |
| Other NDD | spec_share_psychiatrist_c10 | Specialist: Psychiatrist (+10pp) | 0.831104284 | 0.731981528 | 0.935195201 | 950 | 1 | 0.83 (0.73, 0.94) |
| Other NDD | spec_share_psychologist_c10 | Specialist: Psychologist (+10pp) | 1.692150153 | 1.23367806 | 2.469461227 | 3766 | 1 | 1.69 (1.23, 2.47) |
| Other NDD | spec_share_public_health_c10 | Specialist: Public Health (+10pp) | 0.859847699 | 0.749761592 | 0.971416464 | 931 | 1 | 0.86 (0.75, 0.97) |

**Supplementary Table S2c. Autism spectrum disorder: adjusted odds ratios from the individual-level multivariable mixed-effects logistic model. Adjusted odds ratios for ASD with 95% credible intervals are shown for age at diagnosis, sex, registry year, residence, and socioeconomic status categories, with model diagnostics documenting convergence and sampling adequacy.**

| **Disorder** | **term** | **Predictor** | **OR** | **OR_low** | **OR_high** | **OR (95% CrI)** | **ess_bulk** | **r_hat** |
| --- | --- | --- | --- | --- | --- | --- | --- | --- |
| ASD | age_at_diag_c | Age at diagnosis (per 1-year increase) | 0.769511024 | 0.765673071 | 0.773368214 | 0.77 (0.77, 0.77) | 1638 | 1 |
| ASD | sex_std[Male] | Male (ref: Female) | 2.188026509 | 2.127611523 | 2.243416664 | 2.19 (2.13, 2.24) | 2084 | 1 |
| ASD | year_c | Registry year (per 1-year increase) | 1.335091729 | 1.328432931 | 1.341783904 | 1.34 (1.33, 1.34) | 2208 | 1 |
| ASD | location_type[rural] | Rural (ref: Urban) | 0.595115366 | 0.572352625 | 0.617547062 | 0.60 (0.57, 0.62) | 2456 | 1 |
| ASD | SES_group[Other] | SES: Other (ref: baseline) | 1.218962394 | 1.178214315 | 1.254829868 | 1.22 (1.18, 1.25) | 1600 | 1 |
| ASD | SES_group[Out-of-home care] | SES: Out-of-home care (ref: baseline) | 1.05865581 | 1.022243784 | 1.096364822 | 1.06 (1.02, 1.10) | 1698 | 1 |
| ASD | SES_group[Preschool] | SES: Preschool (ref: baseline) | 2.085481993 | 1.948383394 | 2.250157019 | 2.09 (1.95, 2.25) | 2176 | 1 |
| ASD | SES_group[School-age student] | SES: School-age student (ref: baseline) | 0.881614847 | 0.845353835 | 0.921271959 | 0.88 (0.85, 0.92) | 1698 | 1 |

**Supplementary Table S3. Subsampling sensitivity analysis (individual-level ASD model)**

Balanced 1:1 case–control subsampling repeated 50 times with independent seeds, compared with the full-data fit (parsimonious specification). Stable estimates indicate that subsampling did not bias the results.

| **Predictor** | **OR mean (subsample)** | **SD** | **OR range** | **OR full data** |
| --- | --- | --- | --- | --- |
| Registry year | 1.338 | 0.0019 | 1.333–1.342 | 1.350 |
| Male (vs female) | 2.208 | 0.0192 | 2.163–2.245 | 2.139 |
| Age at diagnosis | 0.793 | 0.0010 | 0.790–0.795 | 0.787 |
| Rural (vs urban) | 1.112 | 0.0099 | 1.087–1.129 | 1.122 |

**Supplementary Table S4. Matrix of Spearman correlations of regional annual diagnostic counts between ASD subdiagnoses and other developmental diagnoses, excluding ID and ADHD. For each ASD–non-ASD diagnosis pair, the table reports the median Spearman ρ across regions, the median p-value, and the number of regional annual time series with sufficient non-zero observations for correlation estimation.**

| **Other_code** | **ASD_code** | **rho** | **p** | **n_regions_used** |
| --- | --- | --- | --- | --- |
| F80.0 | F84.0 | -0.68558 | 0.031963 | 13 |
| F80.0 | F84.1 | -0.47448 | 0.111633 | 13 |
| F80.0 | F84.4 | -0.6742 | 0.064037 | 5 |
| F80.0 | F84.5 | 0.136501 | 0.206549 | 8 |
| F80.0 | F84.8 | 0.380441 | 0.24841 | 5 |
| F80.0 | F84.9 | 0.269338 | 0.661328 | 4 |
| F80.1 | F84.0 | -0.26589 | 0.492543 | 8 |
| F80.1 | F84.1 | -0.42985 | 0.255389 | 8 |
| F80.1 | F84.4 | -0.21909 | 0.648521 | 3 |
| F80.1 | F84.5 | -0.50565 | 0.147605 | 5 |
| F80.1 | F84.8 | 0.304982 | 0.118362 | 4 |
| F80.8 | F84.0 | -0.0072 | 0.006267 | 9 |
| F80.8 | F84.1 | 0.347458 | 0.066613 | 9 |
| F80.8 | F84.4 | 0.047673 | 0.46482 | 3 |
| F80.8 | F84.5 | 0.49259 | 0.177789 | 8 |
| F80.8 | F84.8 | 0.452677 | 0.137728 | 5 |
| F80.8 | F84.9 | 0.325707 | 0.285656 | 4 |
| F80.9 | F84.0 | -0.64051 | 0.083754 | 7 |
| F80.9 | F84.1 | -0.17038 | 0.459097 | 7 |
| F80.9 | F84.5 | -0.02476 | 0.215523 | 4 |
| F80.9 | F84.8 | -0.48809 | 0.094692 | 5 |
| F80.9 | F84.9 | 0.25 | 0.628487 | 3 |
| F81.0 | F84.0 | -0.71918 | 0.034317 | 9 |
| F81.0 | F84.1 | -0.72357 | 0.020561 | 9 |
| F81.0 | F84.4 | 0.285394 | 0.080193 | 4 |
| F81.0 | F84.5 | -0.54205 | 0.0234 | 5 |
| F81.0 | F84.8 | -0.17022 | 0.274022 | 4 |
| F81.1 | F84.0 | -0.13363 | 0.078141 | 3 |
| F81.1 | F84.1 | 0 | 0.153574 | 3 |
| F81.1 | F84.4 | 0.258199 | 0.027534 | 3 |
| F81.3 | F84.0 | -0.58017 | 0.061326 | 9 |
| F81.3 | F84.1 | -0.69376 | 0.026064 | 9 |
| F81.3 | F84.4 | 0.054861 | 0.298644 | 4 |
| F81.3 | F84.5 | -0.31069 | 0.167756 | 6 |
| F81.3 | F84.8 | -0.34428 | 0.302222 | 5 |
| F81.8 | F84.0 | 0.349127 | 0.040268 | 6 |
| F81.8 | F84.1 | -0.28678 | 0.111532 | 6 |
| F81.8 | F84.5 | 0.553251 | 0.075015 | 4 |
| F81.8 | F84.8 | 0.412913 | 0.112642 | 4 |
| F81.9 | F84.0 | 0.463817 | 0.057366 | 5 |
| F81.9 | F84.1 | -0.02222 | 0.322217 | 5 |
| F81.9 | F84.5 | 0.278423 | 0.354164 | 3 |
| F81.9 | F84.8 | 0.414151 | 0.143811 | 3 |
| F82 | F84.0 | -0.86603 | 0.040519 | 3 |
| F82 | F84.1 | -0.44721 | 0.133975 | 3 |
| F83 | F84.0 | -0.01367 | 0.067443 | 19 |
| F83 | F84.1 | -0.03983 | 0.024527 | 18 |
| F83 | F84.2 | 0.108195 | 0.02106 | 4 |
| F83 | F84.3 | 0.128489 | 0.637618 | 3 |
| F83 | F84.4 | 0.493771 | 0.136744 | 7 |
| F83 | F84.5 | 0.093822 | 0.057669 | 13 |
| F83 | F84.8 | 0.233507 | 0.466717 | 8 |
| F83 | F84.9 | 0.648936 | 0.137806 | 6 |
| F88 | F84.0 | -0.84932 | 0.000938 | 3 |
| F88 | F84.1 | -0.7 | 0.135082 | 3 |
| F89 | F84.0 | 0.410792 | 0.158302 | 3 |
| F89 | F84.1 | 0.410792 | 0.144704 | 3 |
| F95.0 | F84.0 | -0.75593 | 0.03002 | 5 |
| F95.0 | F84.1 | -0.70711 | 0.083865 | 5 |
| F95.0 | F84.8 | 0.38839 | 0.276393 | 4 |
| F95.1 | F84.0 | -0.81952 | 0.002536 | 3 |
| F95.1 | F84.1 | -0.81258 | 0.002536 | 3 |
| F95.2 | F84.0 | 0.29277 | 0.153564 | 3 |
| F95.2 | F84.1 | 0.29277 | 0.066475 | 3 |

**Supplementary Table S5. Negative-binomial trend models and overdispersion assessment**

Outcome: national annual case count; predictor: calendar year (centred); offset: log child population. A Poisson Pearson dispersion ≫ 1 indicates overdispersion and justifies the negative-binomial (NB2) model.

| **Group** | **NB IRR per year (95% CI)** | **Poisson Pearson dispersion** | **NB dispersion α** |
| --- | --- | --- | --- |
| ASD | 1.273 (1.259–1.286) | 13.7 | 0.0028 |
| ID | 0.993 (0.987–1.000) | 18.1 | 0.0011 |
| Other developmental | 0.921 (0.909–0.934) | 21.5 | 0.0047 |
| ADHD | 0.897 (0.884–0.910) | 1.6 | 0.0010 |

**Supplementary Table S6. Annual percent change (APC) and segmented regression**

APC from a log-linear model of registry-based prevalence; segmented model with breakpoints fixed at the 2019 PMPC restructuring and the 2021 ASD policy expansion.

| **Group** | **Overall APC %/yr (95% CI)** | **p** | **2014–2019** | **2019–2021** | **2021–2024** |
| --- | --- | --- | --- | --- | --- |
| ASD | +27.0 (+25.4 to +28.7) | 0.0000 | +23.5 | +28.0 | +33.7 |
| ID | -0.7 (-1.5 to +0.1) | 0.0854 | -1.8 | -2.0 | +3.3 |
| Other developmental | -7.8 (-9.4 to -6.3) | 0.0000 | -5.3 | -10.4 | -10.2 |
| ADHD | -10.2 (-11.9 to -8.5) | 0.0000 | -10.0 | -17.8 | -1.5 |

**Supplementary Table S7. Per-variable data coverage / missingness**

Completeness of each analytic variable in the cohort (; RECORD).

| **Variable** | **% missing** | **% missing or unknown/unspecified** |
| --- | --- | --- |
| Sex | 0.0 | 0.0 |
| Region | 0.0 | 0.0 |
| Age | 0.0 | 1.1 |
| Diagnosing specialist | 0.0 | 1.1 |
| Ethnicity | 0.0 | 37.7 |
| Citizenship | 12.0 | 12.0 |
| Placement/educational status | 0.0 | 28.6 |
| Urban/rural | 0.0 | 0.0 |

**RECORD reporting checklist**

STROBE items are addressed in the main text; the RECORD-specific items below summarize how this study using routinely collected health data meets the RECORD recommendations.

| **RECORD item** | **Recommendation (abbrev.)** | **Where addressed** |
| --- | --- | --- |
| RECORD 1.1 | Database/registry stated in title/abstract | Title and Abstract; national health register, Ministry of Health. |
| RECORD 1.2 | Type of data, geographic/time coverage | Abstract and sections 2.1-2.3; Kazakhstan, 2014-2024, children aged <=15 years. |
| RECORD 6.1 | Population selection from the database | Sections 2.3-2.4; annual extracts of children on the register, <=15 years, eligible ICD-10 code. |
| RECORD 6.2 | Linkage / code-based selection | Section 2.2.1; ICD-10 primary diagnosis; no cross-year individual linkage because the identifier is not a person key. |
| RECORD 6.3 | Validation of codes/algorithms | Sections 2.2.1 and 2.9; cleaning, date/age recomputation, region harmonization; coding not externally validated in section 4.4. |
| RECORD 7.1 | Variable classification, codes | Section 2.5 and Supplementary Table S2; full ICD-10 grouping listed. |
| RECORD 12.1 | Data-cleaning methods | Sections 2.2.1, 2.7, and 2.9. |
| RECORD 12.2 | Linkage methods | Not applicable; no individual-level linkage key (section 2.2.1 and section 4.4). |
| RECORD 13.1 | Selection of included records | Section 2.4; inclusion by age and valid code; date analyses restricted to valid dates. |
| RECORD 19.1 | Implications of routinely collected data | Sections 4.1-4.4; ascertainment, selection, information bias, and policy effects. |
| RECORD 22.1 | Access to protocol/data/code | Section 2.10; processed data and scripts in repository; raw data restricted. |
